# Carboranyl Derivatives of Rofecoxib with Cytostatic Activity against Human Melanoma and Colon Cancer Cells

**DOI:** 10.1038/s41598-020-59059-3

**Published:** 2020-03-16

**Authors:** Antonio Buzharevski, Svetlana Paskaš, Menyhárt-Botond Sárosi, Markus Laube, Peter Lönnecke, Wilma Neumann, Blagoje Murganić, Sanja Mijatović, Danijelа Maksimović-Ivanić, Jens Pietzsch, Evamarie Hey-Hawkins

**Affiliations:** 1 Institut für Anorganische Chemie, Universität Leipzig, Johannisallee 29, D-04103 Leipzig, Germany; 2 Department of Immunology, Institute for Biological Research “Siniša Stanković”- National Institute of the Republic of Serbia”, Belgrade University, Belgrade, Serbia; 3 Helmholtz-Zentrum Dresden-Rossendorf, Institute of Radiopharmaceutical Cancer Research, Department of Radiopharmaceutical and Chemical Biology, Bautzner Landstrasse 400, D-01328 Dresden, Germany; 4 Technische Universität Dresden, Faculty of Chemistry and Food Chemistry, Mommsenstrasse 4, D-01062 Dresden, Germany

**Keywords:** Structure-based drug design, Drug development, Diversity-oriented synthesis

## Abstract

Owing to the involvement of cyclooxygenase-2 (COX-2) in carcinogenesis, COX-2-selective inhibitors are increasingly studied for their potential cytotoxic properties. Moreover, the incorporation of carboranes in structures of established anti-inflammatory drugs can improve the potency and metabolic stability of the inhibitors. Herein, we report the synthesis of carborane-containing derivatives of rofecoxib that display remarkable cytotoxic or cytostatic activity in the micromolar range with excellent selectivity for melanoma and colon cancer cell lines over normal cells. Furthermore, it was shown that the carborane-modified derivatives of rofecoxib showed different modes of action that were dependent on the cell type.

## Introduction

Nonsteroidal anti-inflammatory drugs (NSAIDs) are among the most widely used therapeutics for the treatment of pain and inflammation^1^. Their molecular target is the enzyme cyclooxygenase (COX), which catalyzes the dioxygenation/cyclization of arachidonic acid to form prostaglandin H2 (PGH2), which is further metabolized to prostaglandins (PGs), which in turn act as mediators of inflammation^2^. The enzyme occurs as two isoforms: a constitutive one, namely COX-1, producing a basic level of PGs, and an inducible one, namely COX-2, activated by inflammatory stimuli^3^. COX-2 expression is also upregulated in multiple human cancers^4,5^. There is a substantial body of evidence that genetic deletion or pharmacological inhibition of COX-2 abrogates tumorigenesis^6–11^. Thus, NSAIDs, in particular COX-2-selective inhibitors, have garnered attention as potential cytostatic drugs. For example, rofecoxib was shown to exhibit excellent potential as a cytotoxic agent^8,12–15^. In addition to inhibition of COX, it was demonstrated that the cytotoxic activity of NSAIDs also involves the inhibition of other cellular targets.

**Figure 1:**
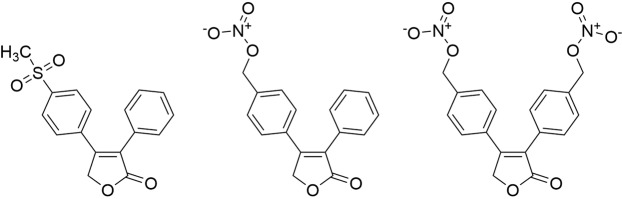
Rofecoxib (left) and NO-releasing rofecoxib analogues (center and right)^24,25^.

Unfortunately, the widespread use of COX-2-selective inhibitors revealed an increased risk for cardiovascular adverse effects in long-term therapy^16,17^. This prompted research into the development of COX-2-selective inhibitors with improved cardiovascular safety profiles. One promising approach is the incorporation of nitric oxide (NO)-releasing moieties into the structures of COX inhibitors, given that NO has vasodilatory activity and inhibits platelet aggregation^18–22^. Moreover, as NO plays an important role in the formation and progression of various cancers^23^, NO-releasing analogues of rofecoxib (Fig. 1) were evaluated for their cytotoxic potency. These compounds retained an inhibitory potential against COX-2 similar to that of rofecoxib but exhibited even higher cytotoxic activity^24,25^.

The highly dynamic field of drug design and synthesis is constantly in search of new pharmacophores. Recently, polyhedral heteroboranes, namely dicarba-*closo*-dodecaboranes (carboranes)^26^, have been increasingly studied as hydrophobic moieties^27,28^. Carboranes are icosahedral clusters composed of ten BH and two CH vertices; different positions of the CH vertices give rise to *ortho* (1,2-), *meta* (1,7-), and *para* isomers (1,12-dic-arba-*closo*-dodecaborane)^29^. On the basis of their hydrophobicity and similarity in dimensions to a phenyl ring (diameter of carborane: 5.25 Å, diameter of a phenyl ring: 4.72 Å), they are regarded as phenyl mimetics^27,28^. Furthermore, carboranes are expected to be resilient to metabolic transformations, and thus may enhance the metabolic stability of known drugs^28,30^. Moreover, one BH vertex can be removed from the icosahedral carborane cluster to generate *nido*-carboranes. These *nido* clusters complement the functional variety of carboranes and, due to their anionic character, can significantly increase the water solubility of the compounds^31,32^.

It has been shown that the incorporation of a carboranyl moiety in place of a phenyl ring in scaffolds of established NSAIDs can lead to compounds with improved activity^33–37^, not only against COX, but also against other targets, such as transthyretin and aldo/keto reductase 1A1^38,39^. Herein, we report the synthesis of five carborane-containing derivatives of rofecoxib as well as their COX inhibitory potential and cytotoxic properties.

## Results and Discussion

### Molecular design and synthesis of rofecoxib analogues

**Figure 2:**
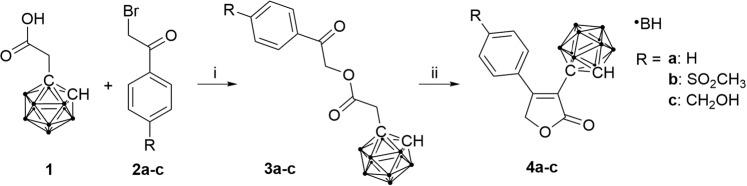
Synthesis of *o*-carboranyl analogues of rofecoxib **4a–c**; (i) Hünigs base (DIPEA), dimethyl sulfoxide (DMSO), 2 h, room temperature; (ii) NaH, anhydrous DMSO, 0 °C.

Metabolic transformation of rofecoxib occurs at the unsubstituted phenyl ring^40^, and hence a carborane cluster was introduced instead at the 3-position of the butenolide ring of rofecoxib (Fig. 2). *o*-Carborane was selected because it can easily be transformed into a *nido* cluster. Moreover, it is known that substituents at the *para* position of the second phenyl ring in rofecoxib contribute to the COX-2 selectivity of the inhibitor and its analogues^41,42^. Therefore, carboranyl analogues with different substituents at this position, including a nitrate moiety as in previously reported NO-releasing rofecoxib prodrugs, were synthesized (Fig. 1)^25^.

Preferably, the carborane cluster is inserted last in the proposed structure to minimize potential loss of the cluster over multiple synthetic steps. This meant that a bond had to be formed between the cluster and the vinyl carbon atom at the 3-position of the butenolide ring. Accordingly, a copper-catalyzed C–C coupling reaction with elevated temperatures and long reaction times was performed^43,44^. However, this approach did not yield the desired product, because the butenolide ring proved to be labile under the required basic conditions at elevated temperatures^45^.

Ultimately, an alternative route was followed that involved a ring-closing reaction of esters **3a–c**, which were obtained by condensation of carboranyl derivative of acetic acid **1** and α-halo-substituted ketones **2a–c** (Fig. 2). Compound **1** was obtained by treating the sodium salt of *o*-carborane with sodium iodoacetate in liquid ammonia. This reaction provides higher yields with *o*-carborane than with *m*- or *p*-carborane^46^. However, as *o*-carborane is highly susceptible to deboronation, precautionary measures must be taken when bases are used in subsequent steps. Thus, in contrast to a previously reported procedure^47^, diisopropylethylamine (DIPEA), a sterically hindered base that acts as a good proton scavenger but exhibits low nucleophilicity, was used in the first step. In the second step, a stronger base, namely NaH, was used because the methylene protons adjacent to the carborane cluster are less acidic than those in the previously reported phenyl analogue of **3a–c**. With this synthetic procedure, esters **3a–c** and ultimately three carboranyl derivatives of rofecoxib, **4a–c**, were synthesized.

**Figure 3:**
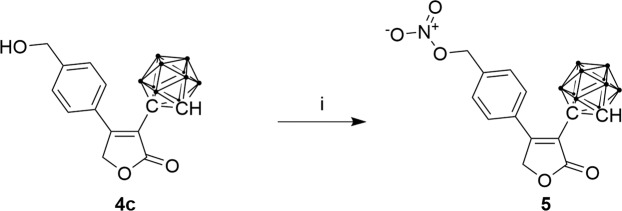
Synthesis of a nitrate derivative of rofecoxib; (i) AgNO_3_, PPh_3_, *N*-bromosuccinimide (NBS), MeCN, 2.5 h at 60 °C.

In an attempt to obtain an NO-releasing NSAID, a nitrate derivative **5** was also synthesized (Fig. 3). With **4c** as starting material, the hydroxyl group was substituted with the better leaving group bromo, which was subsequently replaced by the nucleophilic nitrate ion from AgNO_3_.

**Figure 4:**
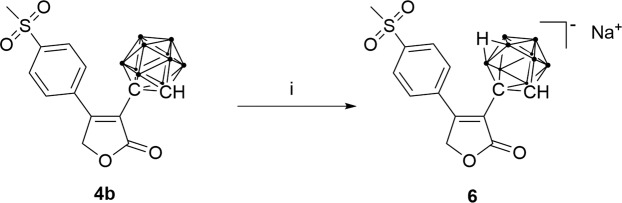
Synthesis of a *nido*-carboranyl analogue of rofecoxib; (i) NaOAc, CH_3_OH, 65 °C, 48 h.

Furthermore, a *nido* derivative of analogue **4b** was obtained by a deboronation reaction in wet methanol with sodium acetate as base (Fig. 4).

All five carborane-containing rofecoxib analogues (**4a–c**, **5**, and **6**) as well as the ester intermediates **3a–c** were fully characterized by 1D and 2D NMR spectroscopy, mass spectrometry, elemental analysis, and X-ray crystallography (Fig. 5; SI, Tables S1–S8, Figs. S1–S8).

**Figure 5:**
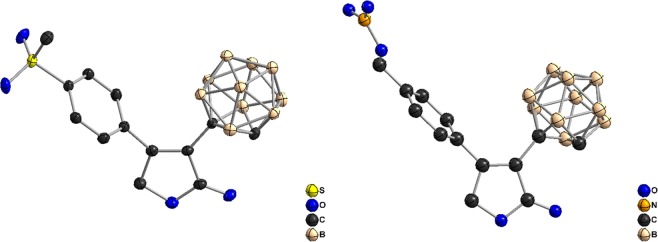
Crystal structures of analogues **4b** (left) and **5** (right). (ORTEP representation: displacement thermal ellipsoids are drawn at 50% probability).

### COX inhibition

**Figure 6:**
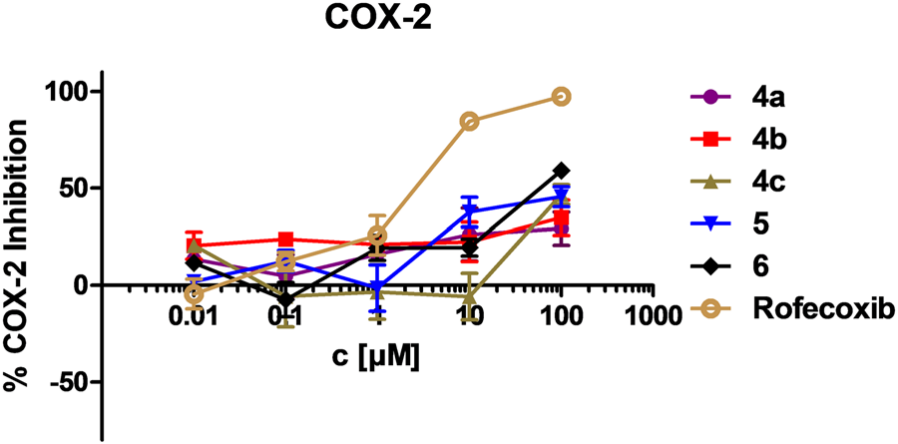
Inhibition of COX-2 by the rofecoxib analogues **4a–c, 5**, and **6**. Error bars indicate standard error mean (SEM) obtained from two measurements at each concentration.

The synthesized compounds were tested *in vitro* for their potential to inhibit ovine COX-1 and human recombinant COX-2. This was done by employing a commercial COX assay (COX Fluorescent Inhibitor Screening Assay Kit, Item No. 700100, Cayman Chemical, Ann Arbor, MI). Of the five synthesized analogues, only *nido* derivative **6** was found to be a weak COX-2-selective inhibitor (IC_50_ (COX-2): 69.63 µM, IC_50_ (COX-1) > 100 µM) (Fig. 6; SI, Fig. S9, Table S9), whereas the other analogues did not exhibit any inhibitory activity (SI, Table S9, Fig. S10).

**Figure 7:**
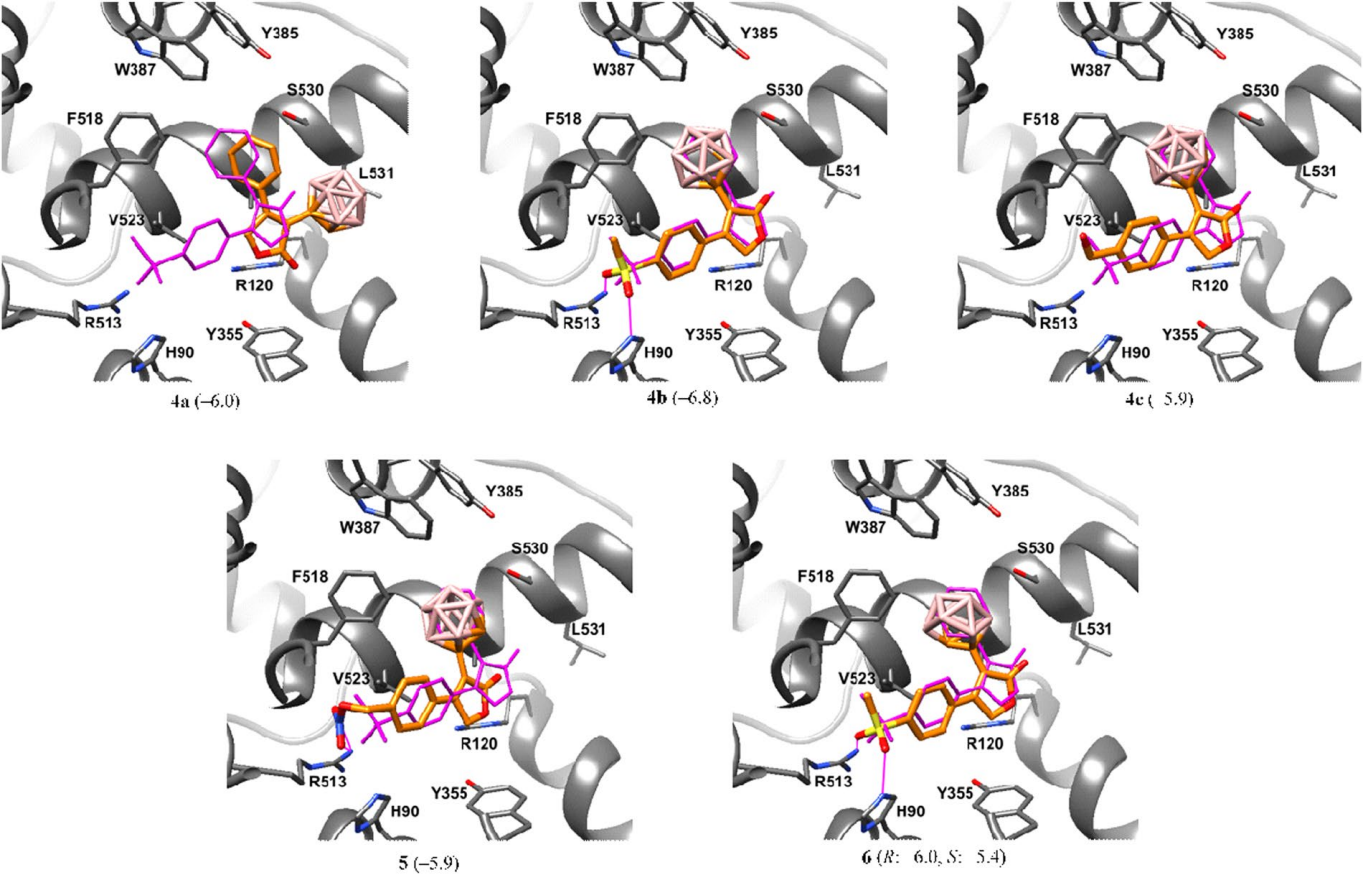
Docked conformations of the carboranyl rofecoxib analogues **4a–c**, **5** and **6**. In parentheses, calculated free energies of binding are given in kcal/mol. Magenta wireframe: superimposed experimental binding pose of rofecoxib from PDB ID: 5KIR. Hydrogen bonds are shown as magenta solid lines. Hydrogen atoms are not shown for clarity.

To explain the inactivity, which likely resulted from a lack of binding to COX, molecular docking studies were performed for the synthesized inhibitors. It was found that all analogues bearing a *p*-substituted benzene ring (**4b**, **4c**, **5**, and **6**) bind in a manner similar to rofecoxib (Fig. 6). The *p*-substituted benzene ring was inserted in the side pocket of the active site of COX-2, while the butenolide ring faced towards the lobby region of the active site of the enzyme with the lactone oxygen atom close to Arg120. Furthermore, the carborane cluster was nestled in the main pocket of the active site and formed hydrophobic interactions with multiple residues. In contrast, analogue **4a** bearing an unsubstituted benzene ring proved to have a different mode of binding than rofecoxib; the phenyl ring pointed towards the main pocket of the active site, while the carborane was oriented towards Leu531 (Fig. 7). This observation coincides with previous reports that polar substituents contribute to the COX-2 selectivity of rofecoxib and its analogues^41,42^. Whereas a binding free energy of –6.9 kcal/mol was calculated for rofecoxib, all inhibitors, except for **4b**, yielded higher binding energies indicating lower binding affinity than rofecoxib. This finding is consistent with the fact that the newly synthesized compounds either do not inhibit COX (**4a–c** and **5**) or are poor inhibitors (**6**, exists as two enantiomers, *R* and *S*) (Fig. 7).

### Antitumor activity

Rofecoxib and its carborane-based analogues were tested against four melanoma (A375, 518A2, B16, B16F10) and four colon cancer (HCT116, SW480, SW620, CT25CL26) cell lines. Both melanoma and colon cancer are tumor types tightly connected with inflammation, while the expression of COX-2 in the selected cell lines was highly variable. Since a lot of discrepancies were found in the literature concerning the expression of this enzyme^5,48–53^, both COX-1 and COX-2 expression was analyzed by Western blot. While COX-1 was uniformly expressed in all tested cell lines, 518A2 and A375 showed higher expression of COX-2 (SI, Fig. S14) in comparison to other tested cell lines. The cells were treated with a wide range of doses of **4a–c**, **5**, and **6** for 48 h and cell viability was determined by MTT (3-(4,5-dimethylthiazol-2-yl)-2,5-diphenyltetrazolium bromide) and CV (crystal violet) assays. The obtained IC_50_ values were similar for both assays (Table 1). Compound **6** was inactive against all cell lines with the exception of 518A2; **4a–c** showed similar efficacy against all tested cell lines (Fig. S12 and S13). Compound **5** caused the highest decrease in cell viability in all of the melanoma cell lines, whereas growth inhibition of the colon cancer cell lines was similar to those of the other rofecoxib analogues (Table 1).

**Table 1:** IC_50_ values for compounds **4a–c**, **5**, and **6** for the growth inhibition of different melanoma and colon cancer cell lines, obtained from MTT and CV assays (three independent experiments each).

		IC_50_ (µM)									
	Compound	4a		4b		4c		5		6	
	Test	MTT	CV	MTT	CV	MTT	CV	MTT	CV	MTT	CV
Cell line*	A375	20.15 ± 1.26	15.95 ± 1.65	16.85 ± 1.35	14.05 ± 0.75	17.95 ± 1.26	10.5 ± 0.75	4.25 ± 1.65	4.7 ± 0.30	>50	>50
	518A2	18.95 ± 1.63	21.4 ± 0.42	13.75 ± 0.21	14.75 ± 0.92	36.35 ± 0.21	41.2 ± 2.12	5.15 ± 0.64	5.5 ± 0.57	36.85 ± 0.49	49.7 ± 0.42
	B16	30.8 ± 2.81	30.55 ± 3.86	18 ± 1.71	22.15 ± 0.65	26.05 ± 2.49	30.8 ± 3.22	10.1 ± 0.40	11.45 ± 1.05	>50	>50
	B16F10	24.3 ± 2.01	25.4 ± 2.41	27.3 ± 2.82	26.6 ± 2.62	28 ± 2.62	26.5 ± 2.62	7.95 ± 3.06	7.65 ± 0.26	>50	>50
	HCT116	13.5 ± 0	12.8 ± 0.5	42.5 ± 1.42	35.9 ± 0	22.15 ± 0.95	19.25 ± 1.46	8.9 ± 0	9.95 ± 0.95	>50	>50
	CT26CL25	42.65 ± 3.17	37.2 ± 2.21	38.1 ± 0.9	36.05 ± 1.25	28.45 ± 2.46	29.25 ± 2.86	31.45 ± 3.27	29.4 ± 2.12	>50	>50
	SW480	22.8 ± 0.7	19 ± 1.91	42.5 ± 0	35.9 ± 0	24.15 ± 1.05	26.8 ± 2.81	16.6 ± 1.01	8.6 ± 0.21	>50	>50
	SW620	24.7 ± 0.42	24.75 ± 0.35	19.25 ± 0.78	21.75 ± 0.64	35.7 ± 0.85	44.35 ± 1.77	10.5 ± 0.14	10.75 ± 0.21	>50	>50

*A375, human melanoma; 518A2, human melanoma; HCT116, human colon carcinoma; SW480, human colon carcinoma; SW620, human colon carcinoma; B16, mouse solid melanoma; B16F10, mouse metastatic melanoma; CT25CL26, mouse colon carcinoma.

**Figure 8:**
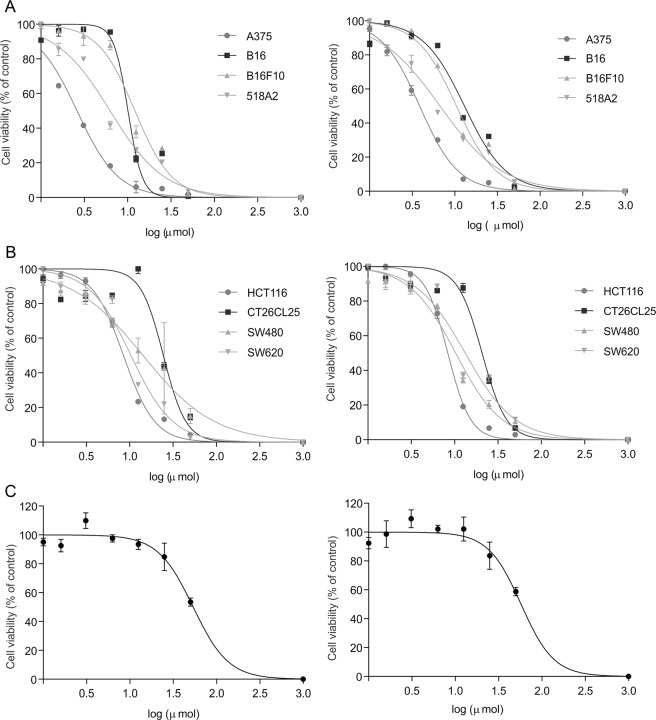
Effect of rofecoxib and analogue **5** on the viability of cancer cells. Melanoma (**A**) and colon cancer cells (**B**) were exposed to **5** or rofecoxib (**C**, only A375 cells) for 48 h and the number of viable cells was analyzed by MTT (left panel) or CV (right panel) assay method. The data are presented as percentage of control ± SD from one representative out of three independent experiments.

The COX-2-expressing melanoma cell lines A375 and 518A2 were the most sensitive to **5**. However, **5** also inhibited the growth of the other tested cell lines, including those with a low level of COX-2 expression. This observation in addition to the weak COX-2 inhibition indicates that the antitumor action of this compound may be mainly COX-2-independent. Moreover, the treatment of A375 cells with rofecoxib did not result in significantly decreased viability, and this suggested that the cytotoxicity of the carborane analogues is related to the carborane substitution (Fig. 8).

In addition to A375 cells, rofecoxib is known to be inactive against COX-2-overexpressing (HCA-7) and COX-2-negative (HCT-116) human colon cancer cell lines^54,55^. On the other hand, rofecoxib was previously shown to inhibit the proliferation of a variety of cancer cell lines, including COX-2-overexpressing and COX-2-negative cells^12,14,15,56,57^. These observations indicate that the antitumor action of rofecoxib can be ascribed to COX-2-independent effects^58^. In addition, it can be inferred that the antitumor potential is not a general feature of rofecoxib but rather defined by some cellular specificity.

A selectivity index was calculated by dividing the IC_50_ of the compound’s cytotoxicity determined for macrophages (SI, Table S10) by that determined for the given cell line. In agreement with the observed experimental effectiveness of the compounds toward the malignant phenotype, the highest selectivity index was calculated for **5** (SI, Table S11,). Thus, further investigations were conducted in which A375 and 518A2 cells were exposed to **5**.

It is known that the A375 cell line has a heterogeneous morphology with a subpopulation of highly metastatic oval cells^59^. Light microscopy of A375 cultures after incubation with **5** revealed not only a decrease in the number of viable cells, but also a more pronounced decrease in oval cells compared to fibroblast-like cells (% of oval vs. fibroblast: 46 ± 2.8 vs. 54 ± 3.2 in the control; 28 ± 2.6* vs. 72 ± 7.4* in the treated cells, *p < 0.05 in comparison to control) (SI, Fig. S11). Thus, it can be concluded that compound **5** affected the two populations in different manners with a stronger effect on the metastatic oval cells. On the other hand, on treatment with **5**, 518A2 cells showed a pattern typical for pronounced cell death, whereby numerous detached, round cells were observed.

**Figure 9:**
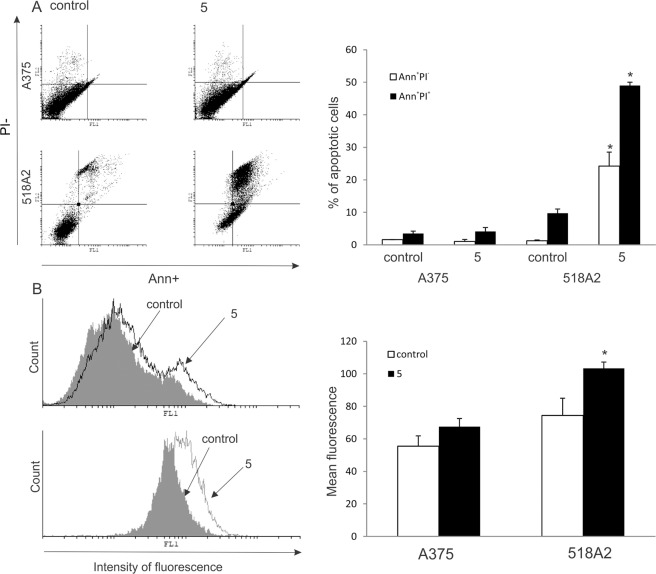
Effect of **5** on apoptosis and caspase activation of A375 and 518A2 cells. Cells were exposed to **5** for 48 h, and apoptosis (**A**) and caspase activation (**B**) were assessed by flow cytometry. The data are presented as a representative dot plot or histogram (left panel) and mean ± SD obtained from three independent experiments (right panel). *p < 0.05 refers to untreated cultures.

Incubation of the A375 cell line with **5** for 48 h did not promote significant apoptosis, as estimated by Ann/PI (AnnexinV-FITC/propidium iodide) double staining (Fig. 9A), and only a slight enhancement of total caspase activity was observed by a pan-caspase inhibitor, ApoStat (Fig. 9B). Conversely, 518A2 cells displayed strong caspase-dependent apoptosis with an elevated percentage of both early Ann^+^PI^−^ and late apoptotic Ann^+^PI^+^ cells (Fig. 9).

**Figure 10:**
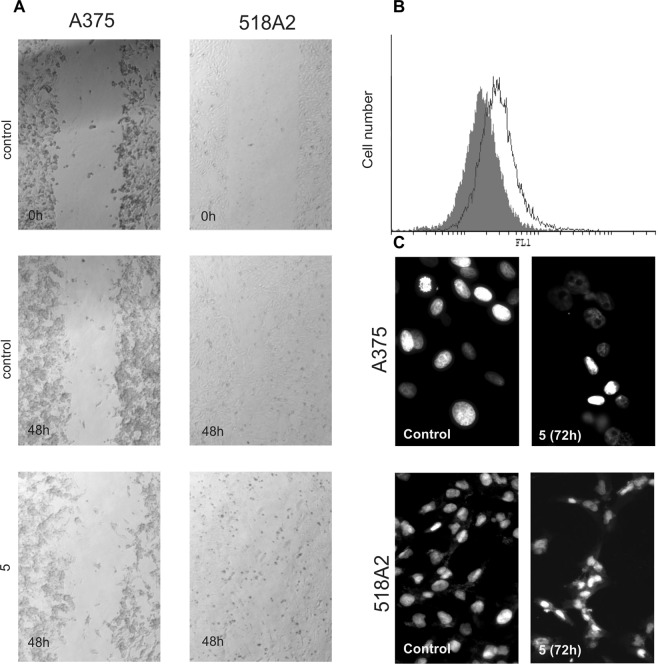
Effect of **5** on the cell proliferation of A375 and 518A2 cells. Cells were exposed to **5**, and cell migration after 48 h was analyzed by light microscopy. (**A**) A375 cell proliferation after 72 h was assessed by flow cytometry. (**B**) Treatments with **5** are presented as black-lined histograms, and controls as gray histograms; the represented histogram was selected out of three repeated experiments. Fluorescence microscopy of DAPI-stained A375 and 518A2 cells after 72 h incubation with **5**, magnification 400× (**C**).

While in the 518A2 cell culture apoptosis was evident, even prolonged incubation of A375 cells with **5** for an additional 24 h (72 h total) was not accompanied by the appearance of apoptotic cells (not shown). Furthermore, the findings of a scratch test performed on both melanoma cell lines treated with **5** showed that 518A2 cells preferentially died while A375 cells revealed inhibited proliferation and migration toward the wound region. It was observed that the wound in the control of the A375 cells decreased from 172 ± 69 μm to 127 ± 13 μm, compared to the treated cells where it stayed at 177 ± 32 μm. In the 518A2 scratch test the wound in the control almost closed (54 ± 12 μm in comparison to 154 ± 2 μm at time 0, p < 0.05), whilst it was impossible to distinguish the edges of the wound in the cells exposed to compound **5** due to the presence of numerous floating dying cells. (Fig. 10A).To confirm this, the cell proliferation rate was determined after 72 h of incubation in the presence of **5** of the carboxyfluorescein succinimidyl ester (CFSE)-stained cells. The obtained data revealed a strong inhibition of A375 cell division manifested through accumulated undivided cells in comparison to a control of untreated cells (% of undivided cells in treated cultures was elevated for 38 ± 8, p < 0.05 in comparison to control) (Fig. 10B). These findings were further confirmed by fluorescence microscopy of 4′,6-diamidino-2-phenylindole (DAPI)-stained cells (Fig. 10C), for which predominantly large nuclei with scarce nucleoli characteristic for interphase were observed on treatment of A375 cells with **5**^60^. In addition, sporadic oblong nuclei with condensed chromatin, typical for apoptosis, were also observed (25 ± 9%). On the other hand in the DAPI-stained 518A2 cells typical apoptotic shrunken nuclei and apoptotic bodies were dominant (60 ± 2%)^61^. The different modes of action on the two melanoma cell lines indicate that cell specific characteristics define the outcome of the treatment with **5**. In comparison, previous studies showed that rofecoxib inhibited proliferation and induced apoptosis as the main mechanism of cell death^12,14,15,57^. Recent data on the compensatory proliferation in response to induced apoptotic cell death in the tumor tissue even favor a nonaggressive approach in the therapy of advanced malignancy^62^.

**Figure 11:**
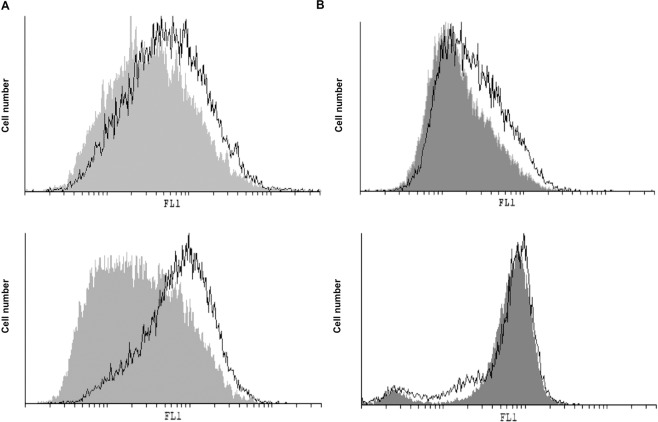
Effect of **5** on intracellular NO and ROS/RNS production in A375 (upper panel) and 518A2 (lower panel) cells. Intracellular NO (**A**) and ROS/RNS (**B**) were measured after 48 h incubation with **5** by flow cytometry. Treatments with **5** are presented as black-lined histograms, controls as gray histograms; the represented histogram was selected out of three repeated experiments.

Given that analogue **5** is designed for NO release, the intracellular release of nitric oxide and production of reactive oxygen species (ROS)/reactive nitrogen species (RNS) was measured by means of redox-sensitive dyes — 4-amino-5-methylamino-2′,7′-difluorofluorescein diacetate (DAF) and dihydrorhodamine-123 (DHR) — after exposure for 48 h (Fig. 11). While a moderate increase of NO was observed in both cell lines (mean fluorescence: 40 ± 7 vs. 60.5 ± 9.1* in A375 cells, and 44.9 ± 5.9 vs. 97.7 ± 1.2* in 518A2 cells, *p < 0.05 refers to untreated cultures), a slight increase in ROS/RNS production was detected only in A375 cultures treated with **5** (mean fluorescence: 22.3 ± 5 vs. 32.67 ± 8* in A375 cells, and 70.7 ± 5.5 vs. 65.6 ± 0.9 in 518A2 cells, *p < 0.05 refers to untreated cultures). It is well documented that highly intensified production of ROS/RNS is connected with extreme cell damage and consequent cell death, whereas moderate ROS production is involved in the regulation of intracellular processes often involved in cell cycle arrest, proliferation, and differentiation^63,64^. Apart from the moderate intracellular NO release, it was important to evaluate whether **5** released NO extracellularly. The production of NO from a nitrate group is a three-electron reduction process that can happen by a number of possible 2e^–^ and 1e^–^ pathways, commonly involving the initial liberation of a nitrite^65^. A common method of detecting the release of NO in the extracellular compartment is the detection of nitrite ions by the Griess reaction, which quantifies the nitrite resulting from the oxidation of NO^66,67^. By this method, no spontaneous NO release from **5** upon incubation in the culture medium or the conditioned culture medium (conditioned culture medium is enriched with soluble cellular products or cell membrane fragments) was detected. Additionally, the NO release was measured in the supernatants of A375 cells exposed to **5** for 48 h, and again no spontaneous NO release was observed in the extracellular compartment. It can be concluded that **5** did not release NO spontaneously in the culture medium or in the extracellular compartment above the detection threshold for the Griess reaction method (approximately 2 μM). Although the introduction of the nitrate moiety resulted in enhanced antitumor potential of **5** compared to the other carboranyl analogues of rofecoxib, this is probably not solely a consequence of NO release. Introduction of a nitrate moiety was also found to be beneficial in the case of rofecoxib. Bocca *et al*. showed that a dinitrate-modified rofecoxib analogue (Fig. 1) had a stronger antiproliferative activity against COX-2-positive HT-29 human colon cancer cells than COX-2-negative SW-480 human colon cancer cells^25^. They demonstrated that, even though COX-2 was inhibited with similar potency, the antitumor potential of the inhibitor was amplified as a consequence of the nitrate modification. Given that analogue **5** also bears a nitrate group and did not inhibit COX-2, further investigations will be needed to better understand the mechanism of action for the observed cytostatic activity.

## Conclusion

We have reported the synthesis of five carborane-containing derivatives (**4a–c**, **5**, and **6**) of rofecoxib in which the phenyl ring in the 3-position in the original structure of rofecoxib was substituted with an *o*-carborane or a *nido*-carborane cluster. All compounds were tested for their COX-inhibitory and cytotoxic activity. Compounds **4a–c** and **5** proved to be inactive against COX, whereas *nido* analogue **6** exhibited weak COX-2 inhibition. Despite that, compounds **4a–c** and **5** exhibited cytotoxic activity against colon cancer and melanoma cell lines. The mode of action of **5** was exemplarily studied on two melanoma cell lines, i.e., A375 and 518A2. While the proliferation and invasiveness of the A375 cell line was greatly inhibited, for the 518A2 cell line caspase-dependent cell death was promoted. These studies provide a foundation for further investigations into analogues of established NSAIDs with improved cytotoxic activities.

### Experimental part

#### Materials and methods

All commercial reagents and solvents were used without further purification. Reactions involving carboranes were carried out under nitrogen atmosphere by using standard Schlenk techniques. Compounds **2a–c** were prepared by a previously reported method^68^. For column chromatography, silica gel 60 Å from ACROS was used. The particle size was in the range of 0.035 to 0.070 mm. To monitor the reaction progress of the syntheses, thin layer chromatography was used. For this purpose, glass plates coated with silica gel 60 F254 from MERCK were used. Carborane-containing areas were stained with a 5% solution of palladium(II) chloride in methanol. All ^1^H, ^13^C, and ^11^B NMR spectra were measured with an ADVANCE DRX 400 spectrometer from BRUKER. The chemical shifts are reported in parts per million (ppm). Quaternary carbon atoms were not always observed because of their long relaxation times. The melting points were determined in glass capillaries with a GALLENKAMP apparatus and are uncorrected. IR spectra were obtained using a FTIR spectrometer (GENESIS ATI, Mattson/Unicam) in the range of 400–4000 cm^−1^ in KBr. The positive or negative mass spectra were recorded with a BRUKER Daltonics APEX II FT-ICR spectrometer. For these measurements, dichloromethane, acetonitrile, methanol, or a mixture of these solvents was used.

#### Carboranyl acetic acid (1)

10 g (69.44 mmol) *o*-carborane, dissolved in 30 mL dry ethyl ether, was added to a stirred solution of 2.7 g (69.23 mmol) sodium amide in 150 mL dry liquid ammonia. The reaction mixture was left to stir at −50 °C under a nitrogen atmosphere. After 1 h of stirring, 14.4 g (69.23 mmol) neat sodium iodoacetate was added. The ammonia was allowed to slowly evaporate from the reaction mixture, leaving the ethereal fraction. 30 mL of *n*-hexane and 30 mL of water were added to the resulting slurry. The organic layer was separated and the aqueous phase was acidified with concentrated HCl. The precipitated carboranyl acetic acid was extracted with diethyl ether. After removal of the organic solvent the crude carboranyl acetic acid was purified by recrystallization from *n*-heptane/toluene (1:1) as colorless needles. The yield of the pure product was 60% (8.41 g, 41.66 mmol). ^1^H NMR (CDCl_3_, 400 MHz): δ = 4.83 (s, 1 H, CH_cluster_), 3.33 (s, 2 H, CH_2_), 3.06–1.45 (m, 10 H, BH_cluster_); ^11^B{^1^H} NMR (CDCl_3_, 128 MHz): δ = −2.0 (s, 1B), −4.8 (s, 1B), −9.1 (s, 2B), −10.5 (s, 2B), −12.0 (s, 2B), −12.7 (s, 2B); ^13^C{^1^H} NMR (CDCl_3_, 100 MHz): δ = 170.8 (COOH), 67.1 (qC_cluster_), 58.6 (CH_cluster_), 40.9 (CH_2_).

#### 2-Phenyl-2-oxoethyl 2-(1,2-dicarba-*closo*-dodecaboranyl)acetate (3a)

1 g (4.94 mmol) carboranyl acetic acid and 1.37 g (6.88 mmol) of **2a** were dissolved in 50 mL DMSO. 0.4 mL DIPEA were added at room temperature to the stirred reaction mixture. The reaction was monitored by TLC (*n*-hexane/ethyl acetate, 2:1). After the starting material was consumed, 50 mL of a 2 M HCl were added with ice cooling. The resulting precipitate was extracted with two 50 mL portions of ethyl acetate. The organic layer was washed with 50 mL water and brine and then dried with MgSO_4_, and the solvent was removed under reduced pressure. The crude product was purified by column chromatography (*n*-hexane/ethyl acetate, 2:1) to afford a pale yellow solid. The yield of the pure product was 80% (1.26 g, 3.95 mmol); mp: 97–98 °C. ^1^H NMR (CDCl_3_, 400 MHz): δ = 7.90 (d, ^3^*J*_HH_ = 8 Hz, 2 H, CH_aromat_), 7.65 (d, ^3^*J*_HH_ = 8 Hz, 1 H, CH_aromat_), 7.51 (t, ^3^*J*_HH_ = 8 Hz, 2 H, CH_aromat_), 5.39 (s, 2 H, CH_2_), 4.71 (s, 1 H, CH_cluster_), 3.46 (s, 2 H, CH_2_), 2.99–1.46 (m, 10 H, BH_cluster_); ^11^B{^1^H} NMR (CDCl_3_, 128 MHz): δ = −2.3 (s, 2B, BH), −5.0 (s, 1B), −9.0 (s, 2B), −10.3 (s, 2B), −12.2 (s, 1B), −12.8 (s, 2B); ^13^C{^1^H} NMR (CDCl_3_, 100 MHz): δ = 190.9 (CO), 166.5 (COO), 134.4 (qC_aromat_), 133.4 (qC_aromat_), 129.0 (CH_aromat_), 127.8 (CH_aromat_), 67.6 (qC_cluster_), 66.9 (COCH_2_O), 59.2 (CH_cluster_), 41.4 (CH_2_COO); IR (KBr): $\tilde{v}=3090$(m; *ν*(C-H_aromat_)), 2566 (m; *ν*(B-H)), 1760 (s; *ν*(C = O)), 1337 (m; *ν*(C-O)), 1178 cm^−1^ (m; *ν*(C-O)); HR-ESI-MS (positive mode, ACN) *m/z* [M + Na]^+^: Calcd. for C_12_H_20_B_10_O_3_Na: 344.2277, found: 344.2218. Elemental analysis: Calcd. for C: 44.99, H: 6.29; Found for C: 45.29, H: 6.23.

#### 2-[4-(Methylsulfonyl)phenyl]-2-oxoethyl 2-(1,2-dicarba-*closo*-dodecaboranyl)acetate (3b)

Starting from 1 g (4.9 mmol) of **1**, the ester **3b** was obtained in a procedure similar to that of ester **3a**. Purification was carried out by column chromatography (*n*-hexane/ethyl acetate, 4:1). The pure product was obtained as a pale yellow solid in 80% yield (1.56 g, 3.92 mmol); mp: 101–102 °C. ^1^H NMR (CDCl_3_, 400 MHz): δ = 8.10 (m, 4 H, CH_aromat_), 5.40 (s, 2 H, CH_2_), 4.59 (s, 1 H, CH_cluster_), 3.47 (s, 2 H, CH_2_), 3.10 (s, 3 H, CH_3_), 3.05–1.6 (m, 10 H, BH_cluster_); ^11^B{^1^H NMR (CDCl_3_, 128 MHz): δ = −2.1 (s, 2B), −4.9 (s, 1B), −9.0 (s, 2B), −10.4 (s, 2B), −12.1 (s, 1B), −12.7 (s, 2B); ^13^C{^1^H} NMR (CDCl_3_, 125 MHz): δ = 190.0 (CO), 166.5 (COO), 145.4 (qC_aromat_), 137.4 (qC_aromat_), 128.7 (CH_aromat_), 128.2 (CH_aromat_), 66.8 (COCH_2_O), 59.1 (CH_cluster_), 44.3 (CH_3_SO_2_), 41.3 (CH_2_COO); IR (KBr): $\tilde{v}=3220$(m; *ν*(C-H_aromat_)), 2570 (m; *ν*(B-H)), 1750 (s; *ν*(C = O)), 1705 (s; *ν*(C = O)), 1315 (m; *ν*(S = O)), 1185 cm^−1^ (m; *ν*(S = O)); HR-ESI-MS (negative mode, CH_3_CN) *m/z* [M-H]^−^: calcd. for C_13_H_21_B_10_O_5_S: 397.2119, found: 397.2130; *m/z* [M-H_2_O-H]^−^: calcd. for C_13_H_19_B_10_O_4_S 379.2007, found: 379.2021. Elemental analysis: Calcd. for C: 39.19, H: 5.57, S: 8.05; Found for C: 38.95, H: 5.63, S: 8.21.

#### 2-[4-(Hydroxymethyl)phenyl]-2-oxoethyl 2-(1,2-dicarba-*closo*-dodecaboranyl)acetate (3c)

Starting from 1 g (4.9 mmol) of **1**, ester **3c** was obtained in a procedure similar to that of ester **3a**. Purification was carried out by column chromatography (*n*-hexane/ethyl acetate, 2:1). The pure product was obtained as a pale yellow solid in 54% yield (0.92 g, 2.62 mmol); mp = 95–96 °C. ^1^H NMR (CDCl_3_, 400 MHz): δ = 7.89 (d, ^3^*J*_*H*H_ = 8 Hz, 2 H, CH_aromat_), 7.51 (d, ^3^*J*_HH_ = 8 Hz, 2 H, CH_aromat_), 5.38 (s, 2 H, CH_2_), 4.81 (s, 2 H, C*H*_2_), 4.71 (s, 1 H, CH_cluster_), 3.46 (s, 2 H, CH_2_), 2.91–1.46 (m, 10 H, BH_cluster_); ^11^B NMR{^1^H} (CDCl_3_, 128 MHz): δ = −2.3 (s, 2B), −5.0 (s, 1B), −9.0 (s, 2B), −10.3 (s, 2B), −12.1 (s, 1B), −12.7 (s, 2B); ^13^C{^1^H} NMR (CDCl_3_, 100 MHz): δ = 190.5 (CO), 166.5 (COO), 147.6 (qC_aromat_), 132.7 (qC_aromat_), 128.1 (CH_aromat_), 126.9 (CH_aromat_), 67.6 (qC_cluster_), 66.9 (COCH_2_O), 64.4 (CH_2_OH), 59.2 (CH_cluster_), 41.4 (CH_2_COO); IR (KBr): $\tilde{v}$ = 3544 (s; *ν*(O-H)), 3068 (m; *ν*(C-H_aromat_)), 2585 (m; *ν*(B-H)), 1736 (s; *ν*(C = O)), 1693 cm^−1^ (s; *ν*(C = O)); HR-ESI-MS (negative mode, ACN) *m/z* [M-H]^−^: Calcd. for C_13_H_21_B_10_O_4_: 349.2443, found: 349.2441; (positive mode, ACN) *m/z* [M + Na]^+^: Calcd. for C_13_H_22_B_10_O_4_Na: 373.2419, found: 373.2389; Elemental analysis: Calcd. for C: 44.56, H: 6.33; Found for C: 44.27, H: 6.33.

#### 4-phenyl-3-(1,2-dicarba-*closo*-dodecaboranyl)furan-2(5 *H*)-one (4a)

**3a** (1 g, 3.12 mmol) was added to a stirred suspension of NaH (60% in mineral oil, 124 mg, 3.12 mmol) in 50 mL of dry DMSO. The reaction mixture was left to stir for 4 h at room temperature under an atmosphere of nitrogen. After completion of the reaction 50 mL of 2 M HCl was added with ice cooling. The precipitate was extracted with 2 × 50 mL ethyl acetate. The organic phase was washed with 50 mL water and brine after which it was dried with anhydrous MgSO_4_, and the organic solvent was removed under reduced pressure. The crude product was purified with column chromatography (*n*-hexane/ethyl acetate, 3:1) that afforded a colorless solid. The yield of the pure product was 74% yield. (0.69 g, 2.3 mmol); mp = 88–89 °C. ^1^H NMR (CDCl_3_, 400 MHz): δ = 7.50 (m, 3 H, CH_aromat_), 7.23 (m, 2 H, CH_aromat_), 5.54 (s, 1 H, CH_cluster_), 4.83 (s, 2 H, CH_2butenolide_), 2.91–1.34 (m, 10 H, BH_cluster_); ^11^B{^1^H} NMR (CDCl_3_, 128 MHz): δ = −2.0 (s, 2B), −3.0 (s, 1B), −8.9 (s, 2B), −10.7 (s, 1B), −11.9 (s, 2B), −13.3 (s, 2B); ^13^C{^1^H} NMR (CDCl_3_, 100 MHz): δ = 130.2 (CH_aromat_), 128.8 (CH_aromat_), 127.2 (CH_aromat_), 73.2 (CH_2 butenolide_), 58.0 (CH_cluster_); IR (KBr): $\tilde{v}$ = 3090 (m; *ν*(C-H_aromat_)), 2566 (m; *ν*(B-H)), 1760 cm^−1^ (s; *ν*(C = O)); HR-ESI-MS (negative mode, ACN) *m/z* [M-H]^−^: Calcd. for C_12_H_17_B_10_O_2_: 302.2195, found: 309.2194; (positive mode, ACN) *m/z* [M + Na]^+^: Calcd. for C_12_H_18_B_10_O_2_Na: 325.2207, found: 325.2189. Elemental analysis: Calcd. for C: 47.67, H: 6.00; Found for C: 47.35, H: 5.95.

#### 4-[4-(methylsulfonyl)phenyl]-3-(1,2-dicarba-*closo*-dodecaboranyl)furan-2(5H)-one (4b)

Starting from 1 g (2.5 mmol) of **3b**, compound **4b** was obtained in a procedure similar to that for compound **4a**. Purification was carried out by column chromatography (*n*-hexane/ethyl acetate, 3:1). The pure product was obtained as a colorless solid in 48% yield (0.45 g, 1.2 mmol); mp = 82–83 °C. ^1^H NMR (CDCl_3_, 400 MHz): δ = 8.09 (d, ^3^*J*_HH_ = 8 Hz, 2 H, CH_aromat_), 7.49 (d, ^3^*J*_HH_ = 8 Hz, 2 H, CH_aromat_), 5.60 (s, 1 H, CH_cluster_), 4.85 (s, 2 H, CH_2butenolide_), 3.14 (s, 3 H, CH_3_), 2.05–1.14 (m, 10 H, BH_cluster_); ^11^B{^1^H} NMR (CDCl_3_, 128 MHz): δ = −2.4 (d, 3B), −8.7 (s, 2B), −11.9 (t, 5B); ^13^C{^1^H} NMR (CDCl_3_, 100 MHz): δ = 163.7 (qC_aromat_), 128.6 (CH_aromat_), 127.9 (CH_aromat_), 77.9 (CH_2butenolide_), 57.9 (CH_cluster_), 44.4 (CH_3_SO_2_); IR (KBr): $\tilde{v}$ = 3091 (m; *ν*(C-H_aromat_)), 2569 (m; *ν*(B-H)), 1739 (s; *ν*(C = O)), 1304 (s; *ν*(S = O)), 1150 cm^−1^ (s; *ν*(S = O)); HR-ESI-MS (negative mode, ACN) *m/z* [M-H]^−^: Calcd. for C_13_H_19_B_10_O_4_S: 379.2007, found: 379.2021; (positive mode, ACN) *m/z* [M + Na]^+^: Calcd. for C_13_H_20_B_10_O_4_SNa: 409.1983, found: 409.1959; Elemental analysis: Calcd for C: 41.04, H: 5.30; Found for C: 41.44, H: 5.27.

#### 4-[4-(hydroxymethyl)phenyl]-3-(1,2-dicarba-*closo*-dodecaboranyl)furan-2(5H)-one (4c)

Starting from 1 g (2.7 mmol) of **3c**, compound **4c** was obtained in a procedure similar to that for compound **4a**. Purification was carried out by column chromatography (*n*-hexane/ethyl acetate, 3:1). The pure product was obtained as a pale yellow solid in 33% yield (0.31 g, 0. 9 mmol); mp: 103–104 °C. ^1^H NMR (CDCl_3_, 400 MHz): δ = 7.50 (d, ^3^*J*_HH_ = 7.5 Hz, 2 H, CH_aromat_), 7.24 (d, ^3^*J*_HH_ = 7.5 Hz, 2 H, CH_aromat_), 5.54 (s, 1 H, CH_cluster_), 4.82 (s, 2 H, CH_2butenolide_), 4.80 (s, 2 H_,_ CH_2_), 1.80 (s, 1 H, OH), 2.86–1.30 (m, 10 H, BH_cluster_); ^11^B{^1^H} NMR (CDCl_3_, 128 MHz): δ = −2.1 (s, 2B), −3.0 (s, 1B), −8.9 (s, 2B), −10.7 (s, 1B), −11.9 (s, 2B), −13.3 (s, 2B); ^13^C{^1^H} NMR (CDCl_3_, 100 MHz): δ = 170.1 (qC_butenolide_), 166.5 (qC_aromat_), 143.2 (qC_aromat_), 127.4 (CH_aromat_), 126.9 (CH_aromat_), 73.2 (CH_2butenolide_), 64.5 (CH_2_OH), 58.0 (CH_cluster_); IR (KBr): $\tilde{v}$ = 3580 (s; *ν*(O-H)), 3092 (m; *ν*(C-H_aromat_)), 2559 (m; *ν*(B-H)), 1756 cm^−1^ (s; *ν*(C = O)); HR-ESI-MS (negative mode, ACN) *m/z* [M-H]^−^: Calcd. for C_13_H_19_B_10_O_3_: 331.2337, found: 331.2351; (positive mode, ACN) *m/z* [M + Na]^+^: Calcd. for C_13_H_20_B_10_O_3_Na: 355.2313, found: 355.2289; Elemental analysis: Calcd. for C: 46.97, H: 6.06; Found for C: 47.33, H: 5.95.

#### Synthesis of 4-[4-(methylnitrooxy)phenyl]-3-(1,2-dicarba-*closo*-dodecaboranyl)furan-2(5 H)-one (5)

AgNO_3_ (0.306 g, 0.0018 mmol) and Ph_3_P (0.284 g, 1.1 mmol) were added to a solution of **4c** (0.3 g, 0.09 mmol) in dry MeCN (20 mL). The mixture was cooled to 5 °C, and *N*-bromosuccinimide (NBS) (0.192 g, 0.0011 mol) was added in portions. Stirring was continued for 1 h at room temperature and then for 2.5 h at 60 °C. Ethyl acetate (30 mL) was added to the mixture, and the precipitate was removed by filtration. The filtrate was washed with 50 mL water and brine, dried with anhydrous MgSO_4_, and concentrated under reduced pressure to give a solid, which was purified by crystallization from diethyl ether/*n*-hexane (1:1) to give **5**. The yield of the pure product was in 43% (0.14 g, 0.03 mmol); mp: 99–100 °C. ^1^H NMR (CDCl_3_, 400 MHz): δ = 7.54 (d, ^3^*J*_HH_ = 7.5 Hz, 2 H, CH_aromat_), 7.31 (d, ^3^*J*_HH_ = 7.5 Hz, 2 H, CH_aromat_), 5.58 (s, 1 H, CH_cluster_), 5.51 (s, 2 H, CH_2_), 4.84 (s, 2 H, CH_2, butenolide_), 2.94–1.39 (m, 10 H, BH_cluster_); ^11^B{^1^H} NMR (CDCl_3_, 128 MHz): δ = −1.9 (s, 2B), −3.1 (s, 1B), −8.8 (s, 2B), −10.8 (s, 1B), −11.9 (s, 2B), −13.2 (s, 2B); ^13^C{^1^H} NMR (CDCl_3_, 100 MHz): δ = 169.8 (CO), 166.5 (qC_aromat_), 134.6 (qC_aromat_), 130.8 (qC_butenolide_), 129.1 (CH_aromat_), 127.8 (CH_aromat_), 120.6 (qC_butenolide_), 73.6 (CH_2_ONO_2_), 73.1 (CH_2butenolide_), 66.7 (qC_cluster_), 57.9 (CH_cluster_); IR (KBr): $\tilde{v}$ = 3095 (m; *ν*(C-H_aromat_)), 2600 (m; *ν*(B-H)), 1751 (s; *ν*(C = O)), 1641 (s; *ν*(N-O)), 1281 cm^−1^ (s; *ν*(N-O)); HR-ESI-MS (negative mode, ACN) *m/z* [M-H]^−^: Calcd. for C_13_H_18_B_10_NO_5_: 376.2188, found: 376.2179; Elemental analysis: Calcd. for C: 41.37, H: 5.07, N: 3.71; Found for C: 41.11, H: 5.07, N: 3.56.

#### Synthesis of *R,S*-7-(2-oxo-4-(4-sulfamoylphenyl)-2,5-dihydrofuran-3-yl)-7,8-dicarba-*nido*-dodeca-hydroundecaborate(–1) sodium (6)

0.5 g (1.31 mmol) **4b** were dissolved in 50 mL methanol and a catalytic amount of sodium acetate was added. The reaction mixture was heated to reflux for 48 h, after which Amberlite IR120 (Na^+^ form) was added. After 1 h of stirring at room temperature the solvent was removed under reduced pressure. This afforded a viscous oil, which was then purified by column chromatography on silica (*n*-hexane/ethyl acetate, 1:1; then *n*-hexane/acetone, 1:1). The obtained yellow oil was re-suspended in methanol and the product was precipitated with CH_2_Cl_2_ as a yellow solid. The yield of the pure product was 98% (0.51 g, 1.29 mmol); mp: 106–107 °C. ^1^H NMR (Acetone[D_6_], 400 MHz): δ = 8.07 (d, ^3^*J*_HH_ = 8 Hz, 2 H, CH_aromat_), 7.88 (d, ^3^*J*_HH_ = 8 Hz, 2 H, CH_aromat_), 4.95 (dd, ^1^*J*_HH_ = 4.65 Hz, 2 H, CH_2_), 3.19 (s, 3 H, CH_3_), 1.5 (s, 1 H, CH_cluster_), 2.58-0.54 (m, 9 H, BH_cluster_) −2.83 (brs, 1 H, *endo*-H). ^11^B{^1^H} NMR (Acetone[D_6_], ppm): δ = −7.8 (s, 1B), −11.0 (s, 1B), −14.8 (s, 1B), −15.4 (s, 1B), −17.2 (s, 1B), −20.8 (s, 1B), −21.6 (s, 1B), −32.0 (s, 1B), −35,2 (s, 1B); ^13^C{^1^H} NMR (Acetone[D_6_], 100 MHz): δ = 170.8 (CO), 154.7 (qC_butenolide_), 141.6 (qC_aromat_), 138.1 (qC_aromat_), 129.2 (CH_aromat_), 127.1 (CH_aromat_), 69.1 (CH_2butenolide_) 43.4; IR (KBr): $\tilde{v}$ = 2518 (m; *ν*(B-H)), 1742 (s; *ν*(C = O)), 1299 (s; *ν*(S = O)), 1159 cm^−1^ (s; *ν*(S = O)); HR-ESI-MS (positive mode, ACN) *m/z* [M + Na]^+^: Calcd. for C_13_H_20_B_9_O_4_SNa_2_: 416.1751, found: 416.1756. Elemental analysis: Calcd. for C: 39.77, H: 5.13, S: 8.17; Found for C: 39.42, H: 5.28, S: 7.91.

## Supplementary information

SI.

## References

[1] Vane JR (1971). Inhibition of prostaglandin synthesis as a mechanism of action for aspirin-like drugs. Nature: New biology.

[2] Needleman P, Truk J, Jakschik BA, Morrison a AR, Lefkowith JB (1986). Arachidonic Acid Metabolism. Annu. Rev. Biochem.

[3] Vane JR, Bakhle YS, Botting RM (1998). Cyclooxygenases 1 and 2. Annu. Rev. Pharmacool. Toxicol..

[4] Fosslien E (2000). Biochemistry of cyclooxygenase (COX)-2 inhibitors and molecular pathology of COX-2 in neoplasia. Crit. Rev. Clin. Lab. Sci..

[5] Denkert C (2001). Expression of Cyclooxygenase 2 in Human Malignant Melanoma. Cancer Res..

[6] Hull MA (2005). Cyclooxygenase-2: how good is it as a target for cancer chemoprevention?. Eur. J. Cancer.

[7] Schönthal A H (2007). Direct non-cyclooxygenase-2 targets of celecoxib and their potential relevance for cancer therapy. British Journal of Cancer.

[8] Patel MI (2005). Celecoxib inhibits prostate cancer growth: evidence of a cyclooxygenase-2-independent mechanism. Clin. Cancer Res..

[9] Riva B (2016). Celecoxib inhibits proliferation and survival of chronic myelogeous leukemia (CML) cells via AMPK-dependent regulation of β-catenin and mTORC1/2. Oncotarget.

[10] Wu GS, Zou SQ, Liu ZR, Tang ZH, Wang JH (2003). Celecoxib inhibits proliferation and induces apoptosis via prostaglandin E2 pathway in human cholangiocarcinoma cell lines. World J. Gastroenterol..

[11] Arber N (2006). Celecoxib for the Prevention of Colorectal Adenomatous Polyps. N. Engl. J. Med..

[12] Vona-Davis L, Riggs DR, Jackson BJ, McFadden DW (2004). Antiproliferative and apoptotic effects of rofecoxib on esophageal cancer *in vitro*(1). J. Surg. Res..

[13] Alam M (2007). Characterization of the effects of cyclooxygenase-2 inhibition in the regulation of apoptosis in human small and non-small cell lung cancer cell lines. Ann. Surg. Oncol..

[14] Mohseni H (2004). COX-2 inhibition demonstrates potent anti-proliferative effects on bladder cancer *in vitro*. J. Surg. Res..

[15] Wood NJ, Quinton NA, Burdall S, Sheridan E, Duffy SR (2007). Exploring the potential chemopreventative effect of aspirin and rofecoxib on hereditary nonpolyposis colorectal cancer-like endometrial cancer cells *in vitro* through mechanisms involving apoptosis, the cell cycle, and mismatch repair gene expression. Int. J. Gynecol. Cancer.

[16] Nissen SE (2016). Cardiovascular Safety of Celecoxib, Naproxen, or Ibuprofen for Arthritis. N. Engl. J. Med..

[17] Nurmohamed Michael T. (2017). Cardiovascular safety of celecoxib, naproxen and ibuprofen. Nature Reviews Rheumatology.

[18] Abdellatif KR (2010). Celecoxib prodrugs possessing a diazen-1-ium-1,2-diolate nitric oxide donor moiety: synthesis, biological evaluation and nitric oxide release studies. Bioorg. Med. Chem. Lett..

[19] Fiorucci S, Antonelli E, Burgaud J-L, Morelli A (2001). Nitric Oxide—Releasing NSAIDs. Drug Saf..

[20] Verdecchia P (2010). Treatment Strategies for Osteoarthritis Patients with Pain and Hypertension. Ther. Adv. Musculoskelet. Dis..

[21] Sarkate AP, Lokwani DK, Patil AA, Bhandari SV, Bothara KG (2011). Synthesis and evaluation of anti-inflammatory, analgesic, ulcerogenicity and nitric oxide-releasing studies of novel ibuprofen analogs as nonulcerogenic derivatives. Med. Chem. Res..

[22] Bechmann N (2015). Novel (pyrazolyl)benzenesulfonamides with a nitric oxide-releasing moiety as selective cyclooxygenase-2 inhibitors. Bioorg. Med. Chem. Lett..

[23] Xu W, Liu LZ, Loizidou M, Ahmed M, Charles IG (2002). The role of nitric oxide in cancer. Cell Res..

[24] Boschi D (2010). Nitrooxymethyl-substituted analogues of rofecoxib: synthesis and pharmacological characterization. Chemistry & biodiversity.

[25] Bocca C, Bozzo F, Ievolella M, Miglietta A (2012). A novel nitro-oxy substituted analogue of rofecoxib reduces human colon cancer cell growth. Mol. Cell. Biochem..

[26] Carter J (1968). Nomenclature of boron compounds. Inorg. Chem..

[27] Scholz M, Hey-Hawkins E (2011). Carbaboranes as Pharmacophores: Properties, Synthesis, and Application Strategies. Chem. Rev..

[28] Valliant JF (2002). The medicinal chemistry of carboranes. Coord. Chem. Rev..

[29] Grimes, R. N. In *Carboranes (Third Edition)* (ed Russell N. Grimes) 1–5 (Academic Press, 2016).

[30] Kallio, M., Callaway, J., Saario, E. & Kahl, S. In *Cancer Neutron Capture Therapy* (ed Yutaka Mishima) 611-613 (Springer US, 1996).

[31] Grimes, R. N. In *Carboranes (Second Edition)* (ed. Russell N. Grimes) 675–699 (Academic Press, 2011).

[32] F. Klanberg, E. L. M., Alfred L. Moye, James C. Carter. In *Inorg. Synth*. (ed. W. L. Jolly) (2007).

[33] Scholz M, Bensdorf K, Gust R, Hey-Hawkins E (2009). Asborin: The Carbaborane Analogue of Aspirin. ChemMedChem.

[34] Scholz M, Blobaum AL, Marnett LJ, Hey-Hawkins E (2011). Synthesis and evaluation of carbaborane derivatives of indomethacin as cyclooxygenase inhibitors. Bioorg. Med. Chem..

[35] Scholz M, Blobaum AL, Marnett LJ, Hey-Hawkins E (2012). Ortho-carbaborane derivatives of indomethacin as cyclooxygenase (COX)-2 selective inhibitors. Bioorg. Med. Chem..

[36] Laube M (2013). 2-Carbaborane-3-phenyl-1H-indoles-synthesis via McMurry reaction and cyclooxygenase (COX) inhibition activity. ChemMedChem.

[37] Neumann W (2015). nido-Dicarbaborate Induces Potent and Selective Inhibition of Cyclooxygenase-2. ChemMedChem.

[38] Scholz M, Steinhagen M, Heiker JT, Beck-Sickinger AG, Hey-Hawkins E (2011). Asborin Inhibits Aldo/Keto Reductase1A1. ChemMedChem.

[39] Johnson SM (2005). Native State Kinetic Stabilization as a Strategy To Ameliorate Protein Misfolding Diseases:  A Focus on the Transthyretin Amyloidoses. Acc. Chem. Res..

[40] Slaughter D (2003). Metabolism of rofecoxib *in vitro* using human liver subcellular fractions. Drug metabolism and disposition: the biological fate of chemicals.

[41] Leblanc Y (1995). Synthesis and biological evaluation of 2,3-diarylthiophenes as selective Cox-2 and Cox-1 inhibitors. Bioorg. Med. Chem. Lett..

[42] Bertenshaw SR (1995). 3,4-diarylthiophenes are selective COX-2 inhibitors. Bioorg. Med. Chem. Lett..

[43] Coult R (1993). C-arylation and C-heteroarylation of icosahedral carboranes via their copper(I) derivatives. J. Organomet. Chem..

[44] Gill WR, Herbertson PL, MacBride JAH, Wade K (1996). Preparation of C-2-pyridyl derivatives of icosahedral carboranes via copper(I) intermediates. J. Organomet. Chem..

[45] Mao B (2002). Examination of rofecoxib solution decomposition under alkaline and photolytic stress conditions. J. Pharm. Biomed. Anal..

[46] Zakharkin LI, Grebennikov AV, Savina LA (1968). Barenylmethyl-β-chlorovinyl ketones. Bulletin of the Academy of Sciences of the USSR, Division of chemical science.

[47] Fox, M. A., Goeta, A. E., Hughes, A. K. & Johnson, A. L. Crystal and molecular structures of the nido-carborane anions, 7,9- and 2,9-C2B9H12−. *J. Chem. Soc., Dalton Trans*., 2132–2141, (2002).

[48] Zhou P (2017). Combination therapy of PKCzeta and COX-2 inhibitors synergistically suppress melanoma metastasis. J. Exp. Clin. Cancer Res..

[49] Valcárcel M (2011). Vascular endothelial growth factor regulates melanoma cell adhesion and growth in the bone marrow microenvironment via tumor cyclooxygenase-2. J. Transl. Med..

[50] Pozzi A (2004). Colon Carcinoma Cell Growth Is Associated with Prostaglandin E2/EP4 Receptor-evoked ERK Activation. J. Biol. Chem..

[51] Lin PC, Lin YJ, Lee CT, Liu HS, Lee JC (2013). Cyclooxygenase-2 expression in the tumor environment is associated with poor prognosis in colorectal cancer patients. Oncol. Lett..

[52] Wasinger C (2014). Autocrine secretion of 15d-PGJ2 mediates simvastatin-induced apoptotic burst in human metastatic melanoma cells. Br. J. Pharmacol..

[53] Che XH (2016). Dual inhibition of COX-2/5-LOX blocks colon cancer proliferation, migration and invasion *in vitro*. Oncol. Rep..

[54] Schiffmann S (2008). The anti-proliferative potency of celecoxib is not a class effect of coxibs. Biochem. Pharmacol..

[55] Kazanov D (2004). Celecoxib But Not Rofecoxib Inhibits the Growth of Transformed Cells *in Vitro*. Clin. Cancer Res..

[56] Zhu FS (2010). Rofecoxib augments anticancer effects by reversing intrinsic multidrug resistance gene expression in BGC-823 gastric cancer cells. J. Dig. Dis..

[57] Alam M (2007). Characterization of the Effects of Cyclooxygenase-2 Inhibition in the Regulation of Apoptosis in Human Small and Non–Small Cell Lung Cancer Cell Lines. Ann Surg Oncol..

[58] Grosch S, Tegeder I, Niederberger E, Brautigam L, Geisslinger G (2001). COX-2 independent induction of cell cycle arrest and apoptosis in colon cancer cells by the selective COX-2 inhibitor celecoxib. FASEB J..

[59] Kozlowski JM, Hart IR, Fidler IJ, Hanna N (1984). A human melanoma line heterogeneous with respect to metastatic capacity in athymic nude mice. J. Natl. Cancer Inst..

[60] Chuang J-Y (2008). Phosphorylation by c-Jun NH2-terminal Kinase 1 Regulates the Stability of Transcription Factor Sp1 during Mitosis. Mol. Biol. Cell.

[61] Ziegler U, Groscurth P (2004). Morphological Features of Cell Death. Physiology.

[62] Mijatovic S (2018). Naturally occurring compounds in differentiation based therapy of cancer. Biotechnol. Adv..

[63] Moloney JN, Cotter TG (2018). ROS signalling in the biology of cancer. Semin. Cell Dev. Biol..

[64] Fionda C, Abruzzese MP, Santoni A, Cippitelli M (2016). Immunoregulatory and Effector Activities of Nitric Oxide and Reactive Nitrogen Species in Cancer. Curr. Med. Chem..

[65] Ignarro LJ (1981). Mechanism of vascular smooth muscle relaxation by organic nitrates, nitrites, nitroprusside and nitric oxide: evidence for the involvement of S-nitrosothiols as active intermediates. J. Pharmacol. Exp. Ther..

[66] Kleinbongard P (2003). Plasma nitrite reflects constitutive nitric oxide synthase activity in mammals. Free Radical Biol. Med..

[67] Sako M, Oda S, Ohara S, Hirota K, Maki Y (1998). Facile Synthesis and NO-Generating Property of 4H-[1,2,5]Oxadiazolo[3,4-d]pyrimidine-5,7-dione 1-Oxides. The Journal of Organic Chemistry.

[68] Mohan RB, Reddy NCG (2013). Regioselective α-Bromination of Aralkyl Ketones Using N-Bromosuccinimide in the Presence of Montmorillonite K-10 Clay: A Simple and Efficient Method. Synth. Commun..

