# Supplementary material for: Carboranyl Derivatives of Rofecoxib with Cytostatic Activity against Human Melanoma and Colon Cancer Cells
Source: Sci Rep. 2020 Mar 16;10:4827. doi: 10.1038/s41598-020-59059-3 (PMC7076013; doi:10.1038/s41598-020-59059-3)
Supplement: Supplementary file 1 — SI. [file 41598_2020_59059_MOESM1_ESM.pdf]

SUPPLEMETARY INFORMATION FOR:

Carboranyl Derivatives of Rofecoxib with Cytostatic Activity Against Human Melanoma and Colon Cancer Cells

Antonio Buzharevski[1], Svetlana Paskaš [2], Menyhárt-Botond Sárosi[1], Markus Laube[3], Peter Lönnecke[1], Wilma Neumann[1], Blagoje Murganić[2], Sanja Mijatović[2], Danijela Maksimović-Ivanić[2], Jens Pietzsch[3,4], Evamarie Hey-Hawkins[1],*

[1]Institut für Anorganische Chemie, Universität Leipzig, Johannisallee 29, D-04103 Leipzig, Germany. Fax: (+49)341-9739319; Correspondence to hey@uni-leipzig.de
[2]Department of Immunology, Institute for Biological Research "Siniša Stanković"- National Institute of the Republic of Serbia", Belgrade University, Serbia
[3]Helmholtz-Zentrum Dresden-Rossendorf, Institute of Radiopharmaceutical Cancer Research, Department of Radiopharmaceutical and Chemical Biology, Bautzner Landstrasse 400, D-01328 Dresden, Germany
[4]Technische Universität Dresden, Faculty of Chemistry and Food Chemistry, Mommsenstrasse 4, D-01062 Dresden, Germany

Table of contents:

1. Crystal structure data 4

Table S1 Crystal data and structure refinement for **3a**. 4

Figure S1 Crystal structure of **3a** 5

Table S2 Crystal data and structure refinement for **3b**. 6

Figure S2 Crystal structure of **3b** 7

Table S3 Crystal data and structure refinement for **3c** 8

Figure S3 Crystal structure of **3c** 9

Table S4 Crystal data and structure refinement for **4a** 10

Figure S4 Crystal structure of **4a** 11

Table S5 Crystal data and structure refinement for **4b** 12

Figure S5 Crystal structure of **4b** 13

Table S6 Crystal data and structure refinement for **4c** 14

Figure S6 Crystal structure of **4c** 15

Table S7 Crystal data and structure refinement for **5** 16

Figure S7 Crystal structure of **5** 17

Table S8 Crystal data and structure refinement for **6** 18

Figure S8 Crystal structure of **6** 19

2. COX inhibition assay 20

Table S9 Measured COX-1 and 2 inhibition values for compounds **4a-c**, **5**, **6** and rofecoxib 20

Figure S9 *Inhibition of COX-2 by compound **6*** 20

Figure S10 *Inhibition ofCOX-1 by rofecoxib analogues **4a-c**, **5** and **6**.* 21

3. Cytotoxicity assays 22

3.1. Reagents and cells. 22

3.2. Cell viability tests. 22

3.3. AnnexinV-FITC/PI and caspase detection. 23

3.4. Cell staining with CFSE. 23

3.5. Wound healing test. 23

3.6. Measurement of intracellular nitric oxide 23

3.7. Measurement of intracellular reactive oxygen (ROS) and nitrogen (RNS) species 24

3.8. Nitrite detection 24
3.9. Western blot. 25
3.10. Statistics 25
3.11. Molecular docking studies 25
Table S10 IC_{50} values for the cytotoxicity of **4a-c**, **5** and **6** for isolated macrophages measured by the crystal violet (CV) test. 26
Table S11 *Selectivity index values of celecoxib in melanoma and colon cancer cell lines.* 26
Figure S11 Light microscopy of cells after incubation with **5** for 48 h 27
Figure S12 Influence of rofecoxib analogues **4a-c** on melanoma viability**.**. 28
Figure S13 Influence of rofecoxib analogues **4a-c** on viability of colon cancer cell lines.. 29
Figure S14 *COX-1 and COX-2 expression.* 30
*^{1}H NMR spectrum of compound **4a*** 30
*^{1}H MNR spectrum of compound **4b*** 31
*^{1}H NMR spectrum of compound **4c*** 32
*^{1}H NMR spectrum of compound **5*** 33
*^{1}H NMR spectrum of compound **6*** 34
References: 34

1. Crystal structure data

The data were collected on a Gemini diffractometer (Rigaku Inc.) using Mo-Kα radiation (λ = 71.073 pm) and ω-scan rotation. Data reduction was performed with CrysAlis Pro[1] including the program SCALE3 ABSPACK for empirical absorption correction. All structures were solved by dual-space methods[2] and the refinement was performed with SHELXL-2018.[3] With the exception of some disordered parts of a structure, all non-hydrogen atoms were refined with anisotropic thermal parameters. Carborane carbon atoms are localized with a bond length and displacement parameter analysis. CCDC 1875620 (**3a**), 1875621 (**3b**), 1875622 (**3c**), 1875623 (**4a**), 1875624 (**4b**), 1875625 (**4c**), 1875626 (**5**) and 1875627 (**6**) contains the supplementary crystallographic data for this paper. These data can be obtained free of charge via https://summary.ccdc.cam.ac.uk/structure-summary-form (or from the Cambridge Crystallographic Data Centre, 12 Union Road, Cambridge CB2 1EZ, UK; fax: (+44)1223-336-033; or deposit@ccdc.cam.uk).

***Table S1** Crystal data and structure refinement for **3a**.*

Empirical formula	$C_{12}H_{20}B_{10}O_3$
Formula weight	320.38
Temperature	130(2) K
Wavelength	71.073 pm
Crystal system	Orthorhombic
Space group	*P*bca
Unit cell dimensions	a = 1132.36(2) pm<br>α = 90°<br>b = 990.85(2) pm<br>β = 90°<br>c = 3093.96(6) pm<br>γ = 90°
Volume	3.4714(1) nm^3
Z	8
Density (calculated)	1.226 Mg/m^3
Absorption coefficient	0.072 mm^{-1}
F(000)	1328
Crystal size	0.4 x 0.25 x 0.2 mm^3
Theta range for data collection	2.229 to 32.029°
Index ranges	-16 ≤ h ≤ 16, -14 ≤ k ≤ 13, -45 ≤ l ≤ 45

Reflections collected	40321
Independent reflections	5761 [R(int) = 0.0408]
Completeness to theta = 30.510°	100.00%
Absorption correction	Semi-empirical from equivalents
Max. and min. transmission	1 and 0.96396
Refinement method	Full-matrix least-squares on F^2
Data / restraints / parameters	5761 / 0 / 306
Goodness-of-fit on F^2	1.065
Final R indices [I>2sigma(I)]	R1 = 0.0542, wR2 = 0.1125
R indices (all data)	R1 = 0.0755, wR2 = 0.1218
Largest diff. peak and hole	0.332 and -0.200 e·Å^{-3}

*All H atoms were located on difference Fourier maps calculated at the final stage of the structure refinement.

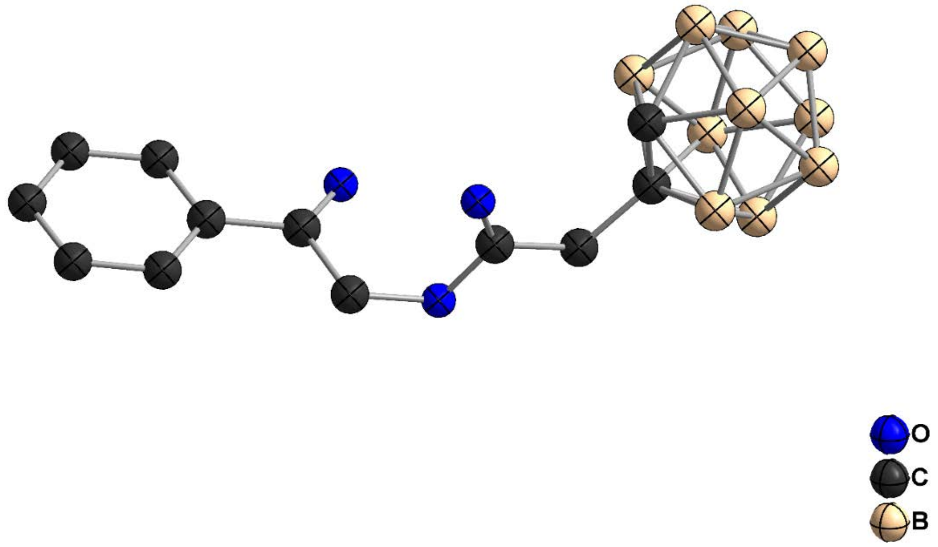

Figure S1 *Crystal structure of* ***3a***

Table S2 *Crystal data and structure refinement for* ***3b***.

Empirical formula	$C_{13}H_{22}B_{10}O_5S$
Formula weight	398.46
Temperature	130(2) K
Wavelength	71.073 pm
Crystal system	Triclinic
Space group	$P\bar{1}$
Unit cell dimensions	a = 970.20(5) pm<br>α = 68.509(4)°<br>b = 1030.33(5) pm<br>β = 85.488(4)°<br>c = 1172.95(5) pm<br>γ = 66.730(5)°
Volume	0.99925(9) nm^3
Z	2
Density (calculated)	1.324 Mg/m^3
Absorption coefficient	0.185 mm^{-1}
F(000)	412
Crystal size	0.30 x 0.20 x 0.05 mm^3
Theta range for data collection	2.292 to 37.771°
Index ranges	$-16 \leq h \leq 16$, $-17 \leq k \leq 17$, $-19 \leq l \leq 19$
Reflections collected	33839
Independent reflections	10171 [R(int) = 0.0345]
Completeness to theta = 36.320°	100.00%
Absorption correction	Semi-empirical from equivalents
Max. and min. transmission	1.00000 and 0.97785
Refinement method	Full-matrix least-squares on F^2
Data / restraints / parameters	10171 / 0 / 350
Goodness-of-fit on F^2	1.028
Final R indices [I>2sigma(I)]	R1 = 0.0526, wR2 = 0.1201
R indices (all data)	R1 = 0.0818, wR2 = 0.1334
Largest diff. peak and hole	0.731 and -0.342 e·$Å^{-3}$

* All H atoms were located on difference Fourier maps calculated at the final stage of the structure refinement.

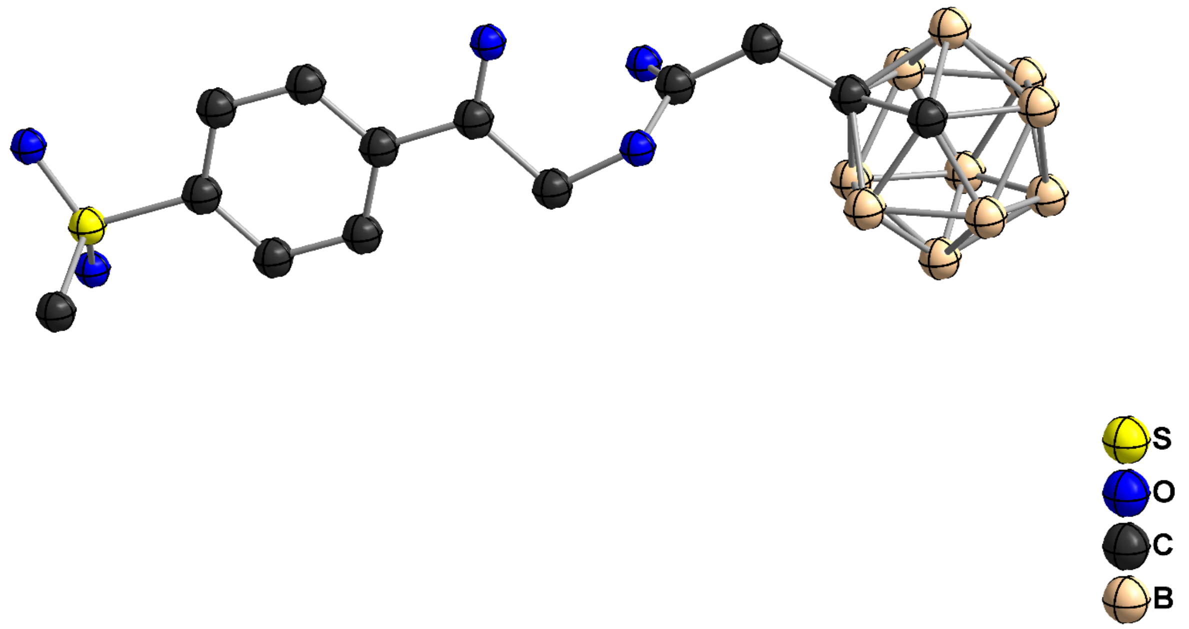

Figure S2 *Crystal structure of* ***3b***

Table S3** Crystal data and structure refinement for **3c

Empirical formula	$C_{13}H_{22}B_{10}O_4$
Formula weight	350.4
Temperature	130(2) K
Wavelength	71.073 pm
Crystal system	Monoclinic
Space group	$P2_1/c$
Unit cell dimensions	a = 2114.79(3) pm<br>α= 90°<br>b = 709.420(10) pm<br>β= 104.377(2)°<br>c = 1244.37(2) pm<br>γ = 90°
Volume	1.80843(5) nm^3
Z	4
Density (calculated)	1.287 Mg/m^3
Absorption coefficient	0.079 mm^{-1}
F(000)	728
Crystal size	0.40 x 0.40 x 0.10 mm^3
Theta range for data collection	1.988 to 34.487°
Index ranges	$-33 \leq h \leq 33$, $-11 \leq k \leq 11$, $-19 \leq l \leq 19$
Reflections collected	42017
Independent reflections	7372 [R(int) = 0.0303]
Completeness to theta = 33.140°	100.00%
Absorption correction	Semi-empirical from equivalents
Max. and min. transmission	1.00000 and 0.98878
Refinement method	Full-matrix least-squares on F^2
Data / restraints / parameters	7372 / 0 / 332
Goodness-of-fit on F^2	1.039
Final R indices [I>2sigma(I)]	R1 = 0.0442, wR2 = 0.1087
R indices (all data)	R1 = 0.0602, wR2 = 0.1167
Largest diff. peak and hole	0.402 and -0.230 $e \cdot Å^{-3}$

*All H atoms were located on difference Fourier maps calculated at the final stage of the structure refinement. With intermolecular OH-O hydrogen donor-acceptor bonds zig-zag chains are formed along (0 1 0).

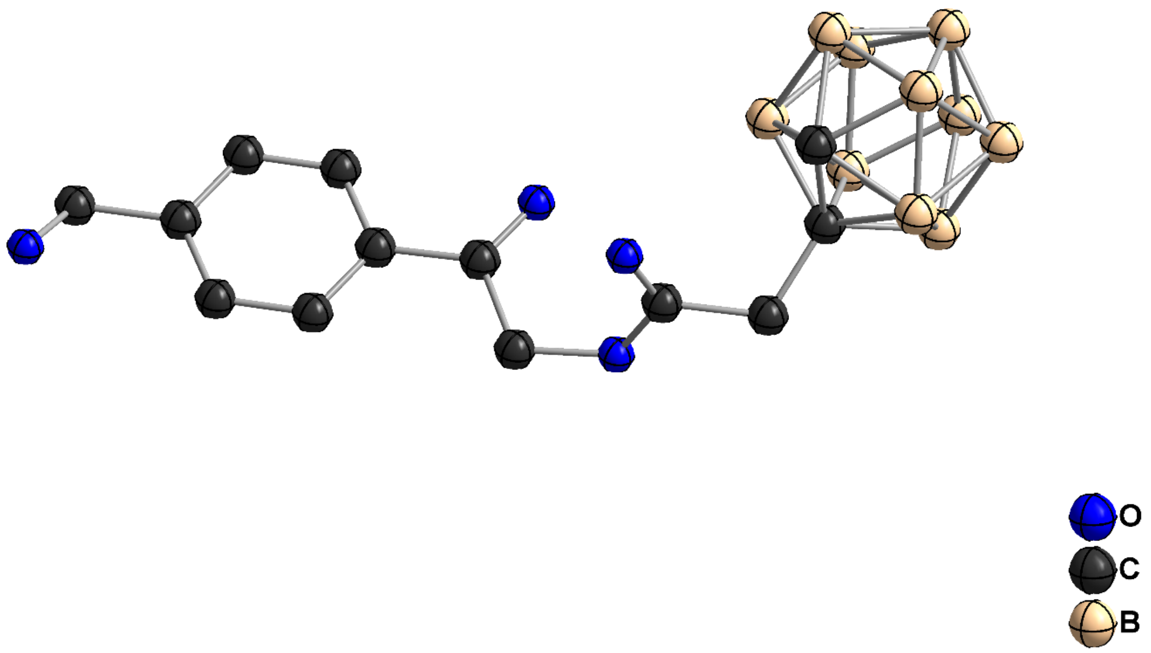

Figure S3 *Crystal structure of* ***3c***

Table S4 *Crystal data and structure refinement for* ***4a***

Empirical formula	$C_{12}H_{18}B_{10}O_2$
Formula weight	302.36
Temperature	130(2) K
Wavelength	71.073 pm
Crystal system	Orthorhombic
Space group	$P2_12_12_1$
Unit cell dimensions	a = 653.40(3) pm<br>α= 90°<br>b = 1324.35(5) pm<br>β= 90°<br>c = 1873.13(8) pm<br>γ = 90°
Volume	1.6209(1) nm^3
Z	4
Density (calculated)	1.239 Mg/m^3
Absorption coefficient	0.069 mm^{-1}
F(000)	624
Crystal size	0.40 x 0.15 x 0.03 mm^3
Theta range for data collection	2.175 to 29.047°
Index ranges	$-8 \le h \le 8$, $-16 \le k \le 17$, $-23 \le l \le 25$
Reflections collected	9852
Independent reflections	3747 [R(int) = 0.0370]
Completeness to theta = 26.375°	99.90%
Absorption correction	Semi-empirical from equivalents
Max. and min. transmission	1.00000 and 0.85312
Refinement method	Full-matrix least-squares on F^2
Data / restraints / parameters	3747 / 0 / 289
Goodness-of-fit on F^2	1.031
Final R indices [I>2sigma(I)]	R1 = 0.0455, wR2 = 0.0913
R indices (all data)	R1 = 0.0691, wR2 = 0.1007
Absolute structure parameter	0.3(7) Without significant information for CHBO
Largest diff. peak and hole	0.175 and -0.199 e $Å^{-3}$

*All H atoms were located on difference Fourier maps calculated at the final stage of the structure refinement.

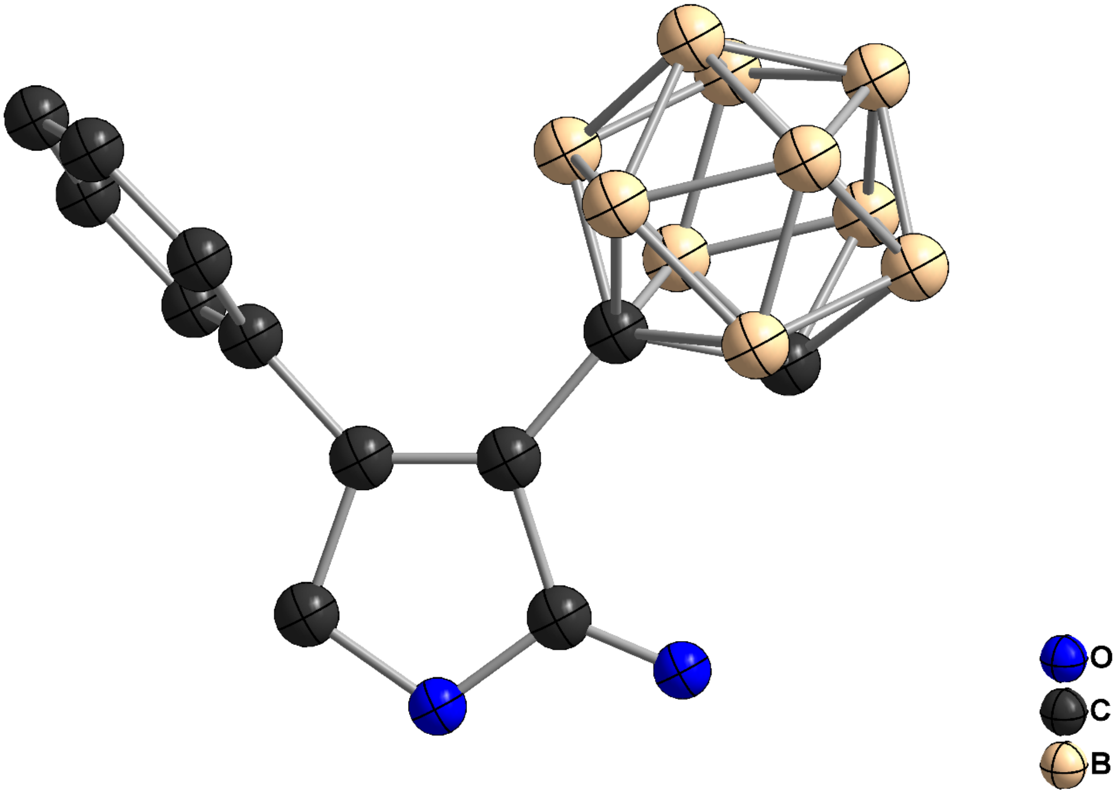

Figure S4 *Crystal structure of* ***4a***

Table S5 *Crystal data and structure refinement for* ***4b***

Empirical formula	$C_{13}H_{20}B_{10}O_4S$
Formula weight	380.45
Temperature	130(2) K
Wavelength	71.073 pm
Crystal system	Orthorhombic
Space group	P c a 2_1
Unit cell dimensions	a = 1259.22(2) pm<br>α= 90°<br>b = 1290.45(2) pm<br>β= 90°<br>c = 1190.14(2) pm<br>γ = 90°
Volume	1.93393(5) nm^3
Z	4
Density (calculated)	1.307 Mg/m^3
Absorption coefficient	0.184 mm^{-1}
F(000)	784
Crystal size	0.35 x 0.30 x 0.20 mm^3
Theta range for data collection	2.260 to 32.601°
Index ranges	$-18 \le h \le 19$, $-19 \le k \le 18$, $-18 \le l \le 17$
Reflections collected	26285
Independent reflections	6479 [R(int) = 0.0365]
Completeness to theta = 30.510°	100.00%
Absorption correction	Semi-empirical from equivalents
Max. and min. transmission	1.00000 and 0.98885
Refinement method	Full-matrix least-squares on F^2
Data / restraints / parameters	6479 / 1 / 333
Goodness-of-fit on F^2	1.029
Final R indices [I>2sigma(I)]	R1 = 0.0366, wR2 = 0.0792
R indices (all data)	R1 = 0.0451, wR2 = 0.0832
Absolute structure parameter	0.02(3)
Largest diff. peak and hole	0.213 and -0.265 e $Å^{-3}$

*All H atoms were located on difference Fourier maps calculated at the final stage of the structure refinement.

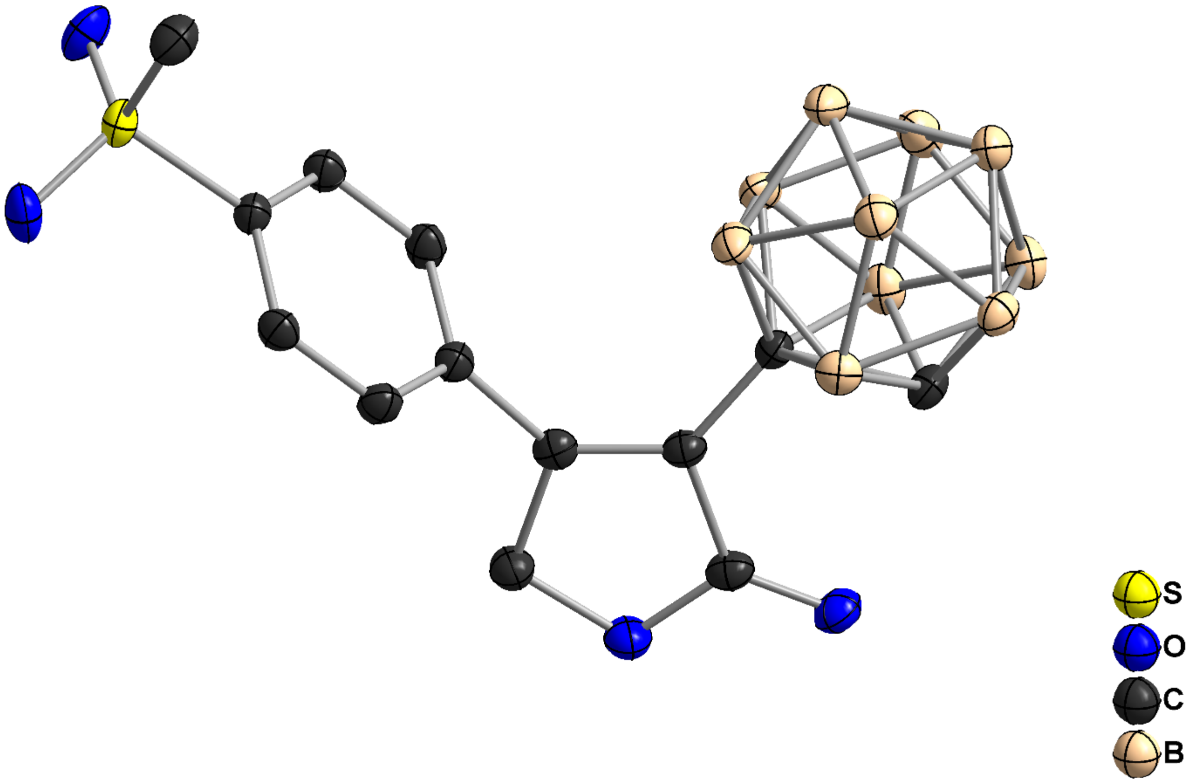

Figure S5 *Crystal structure of* ***4b***

Table S6** Crystal data and structure refinement for **4c

Empirical formula	$C_{13}H_{20}B_{10}O_3$
Formula weight	332.39
Temperature	130(2) K
Wavelength	71.073 pm
Crystal system	Orthorhombic
Space group	$P\ 2_1\ 2_1\ 2_1$
Unit cell dimensions	a = 651.06(5) pm<br>α= 90°<br>b = 1192.8(1) pm<br>β= 90°<br>c = 2247.1(1) pm<br>γ = 90°
Volume	1.7450(2) nm^3
Z	4
Density (calculated)	1.265 Mg/m^3
Absorption coefficient	0.075 mm^{-1}
F(000)	688
Crystal size	0.15 x 0.10 x 0.05 mm^3
Theta range for data collection	1.813 to 28.091°
Index ranges	$-8 \le h \le 7$, $-14 \le k \le 14$, $-28 \le l \le 28$
Reflections collected	12938
Independent reflections	3631 [R(int) = 0.1293]
Completeness to theta = 25.350°	100.00%
Absorption correction	Semi-empirical from equivalents
Max. and min. transmission	1.00000 and 0.58514
Refinement method	Full-matrix least-squares on F^2
Data / restraints / parameters	3631 / 0 / 236
Goodness-of-fit on F^2	0.979
Final R indices [I>2sigma(I)]	R1 = 0.0731, wR2 = 0.1036
R indices (all data)	R1 = 0.1928, wR2 = 0.1373
Absolute structure parameter	0.1(10) Without significant information for CHBO
Largest diff. peak and hole	0.226 and -0.250 e $Å^{-3}$

*All hydrogen atoms were calculated on idealized positions. The compound shows an extreme tendency for dendritic growth and after several attempts, only a very small pure single crystal could be isolated from the cluster.

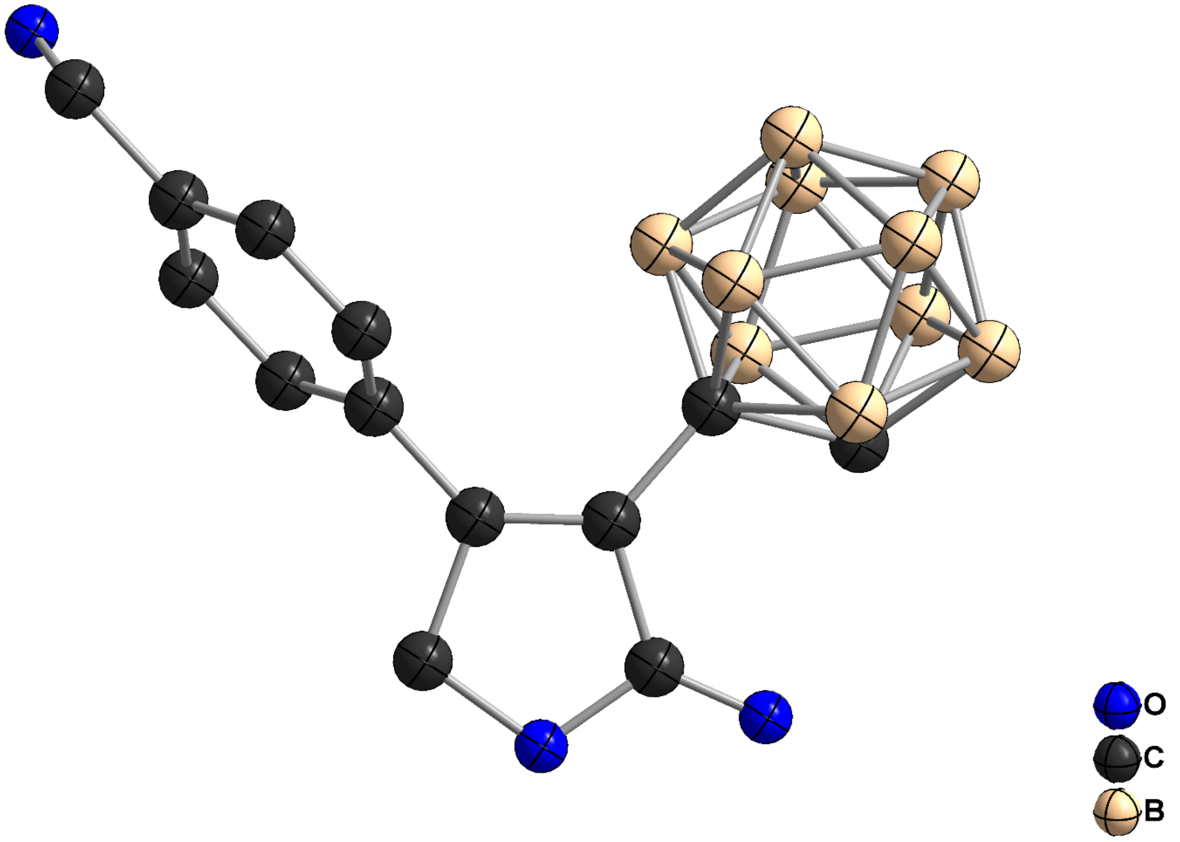

Figure S6 *Crystal structure of* ***4c***

Table S7** Crystal data and structure refinement for **5

Empirical formula	$C_{13}H_{19}B_{10}NO_5$
Formula weight	377.39
Temperature	130(2) K
Wavelength	71.073 pm
Crystal system	Triclinic
Space group	$P\bar{1}$
Unit cell dimensions	a = 792.36(3) pm<br>α= 108.912(4)°<br>b = 982.22(4) pm<br>β= 96.465(3)°<br>c = 1335.70(5) pm<br>γ = 102.620(3)°
Volume	0.94053(7) nm^3
Z	2
Density (calculated)	1.333 Mg/m^3
Absorption coefficient	0.087 mm^{-1}
F(000)	388
Crystal size	0.30 x 0.25 x 0.20 mm^3
Theta range for data collection	2.277 to 32.461°
Index ranges	$-11 \le h \le 11$, $-14 \le k \le 14$, $-20 \le l \le 19$
Reflections collected	16515
Independent reflections	6214 [R(int) = 0.0213]
Completeness to theta = 30.510°	100.00%
Absorption correction	Semi-empirical from equivalents
Max. and min. transmission	1.00000 and 0.99375
Refinement method	Full-matrix least-squares on F^2
Data / restraints / parameters	6214 / 0 / 338
Goodness-of-fit on F^2	1.024
Final R indices [I>2sigma(I)]	R1 = 0.0426, wR2 = 0.1028
R indices (all data)	R1 = 0.0563, wR2 = 0.1113
Largest diff. peak and hole	0.357 and -0.258 e $Å^{-3}$

*All H atoms were located on difference Fourier maps calculated at the final stage of the structure refinement.

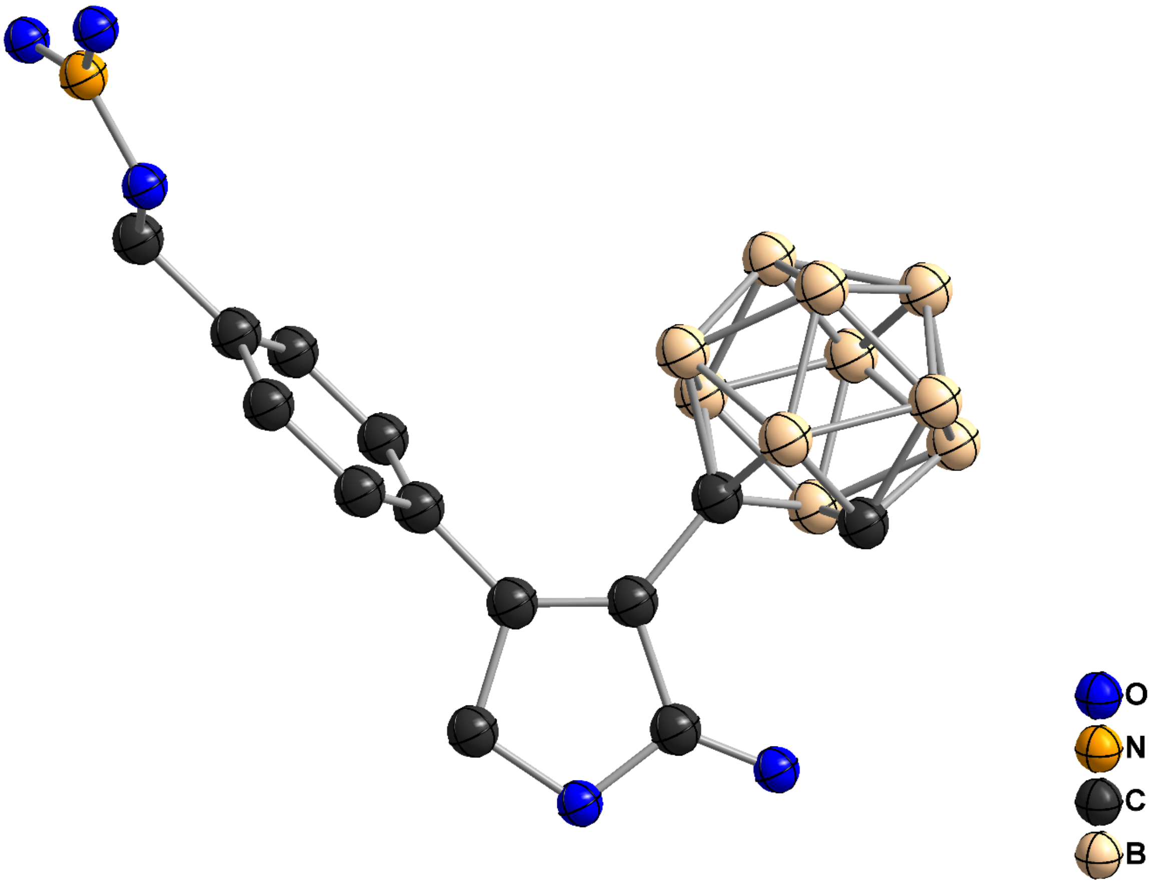

Figure S7 *Crystal structure of* ***5***

Table S8** Crystal data and structure refinement for **6

Empirical formula	$C_{50}H_{92}B_{18}Na_2O_{20}S_2$
Formula weight	1317.91
Temperature	130(2) K
Wavelength	71.073 pm
Crystal system	Orthorhombic
Space group	*C* c c a
Unit cell dimensions	a = 2974.2(1) pm<br>α= 90°<br>b = 4552.4 (1) pm<br>β= 90°<br>c = 1009.95(3) pm<br>γ = 90°
Volume	13.6743(7) nm^3
Z	8
Density (calculated)	1.280 Mg/m^3
Absorption coefficient	0.158 mm^{-1}
F(000)	5568
Crystal size	0.40 x 0.20 x 0.03 mm^3
Theta range for data collection	2.176 to 27.137°
Index ranges	$-35 \le h \le 37$, $-57 \le k \le 58$, $-12 \le l \le 12$
Reflections collected	39132
Independent reflections	7120 [R(int) = 0.0717]
Completeness to theta = 25.350°	99.90%
Absorption correction	Semi-empirical from equivalents
Max. and min. transmission	1.00000 and 0.95321
Refinement method	Full-matrix least-squares on F^2
Data / restraints / parameters	7120 / 408 / 679
Goodness-of-fit on F^2	1.048
Final R indices [I>2sigma(I)]	R1 = 0.0660, wR2 = 0.1498
R indices (all data)	R1 = 0.1195, wR2 = 0.1733
Largest diff. peak and hole	0.323 and -0.323 e $Å^{-3}$

*The structure is highly disordered. Excluding H2Y and H2FY all hydrogen atoms were calculated on idealized positions. Carborane carbon atoms could be localized only for the major 70% fraction of the disordered carborane moiety with a reasonable accuracy.

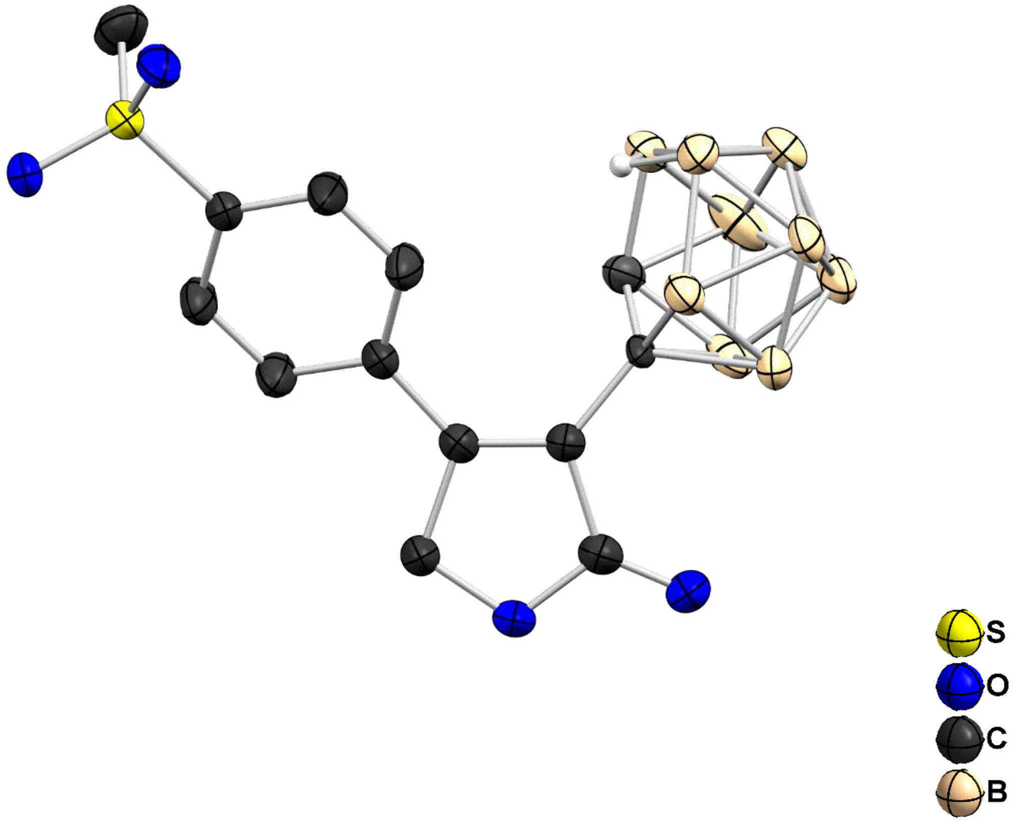

Figure S8 *Crystal structure of* ***6***

2. COX inhibition assay

Table S9 *Measured COX-1 and 2 inhibition values for compounds **4a-c**, **5**, **6** and rofecoxib*

Compound	IC_{50} (μM)	
	COX-1	COX-2
**4a**	>100	>100
**4b**	>100	>100
**4c**	>100	>100
**5**	>100	>100
**6**	>100	69.63
**Rofecoxib**	>100	2.40
**Celecoxib****	>100	0.04±0.02 (n=6)

*All compounds were measured with the Cytation 5 plate reader and the "COX Fluorescent Inhibitor Screening Assay Kit, Item No. 700100 (Cayman chemicals, USA)" according to the manufacturer's instructions.** Celecoxib served as reference. The pIC_{50} ($pIC_{50} = -log_{10} (IC_{50} [M])$) was found be 7.48±0.27 (mean±SD, n=6; IC_{50} =38.1±20.7 nM) with an inter-assay coefficient of variation of 3.6%.

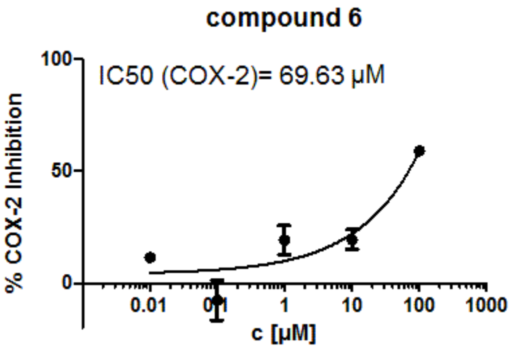

Figure S9 *Inhibition of COX-2 by compound **6**. Error bars indicate standard error mean (SEM) obtained from two measurements at each concentration.*

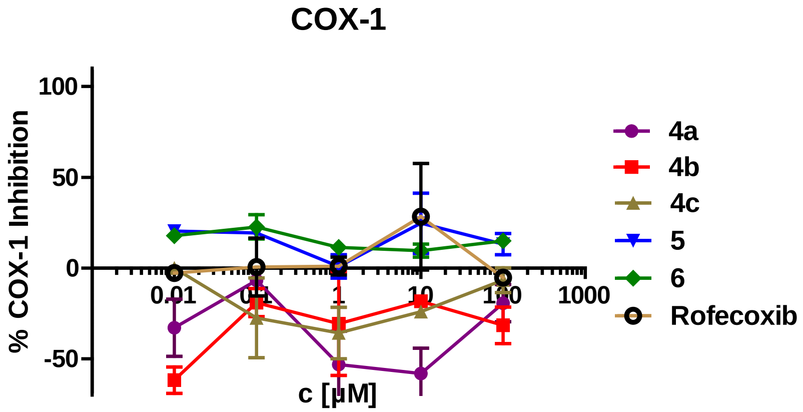

Figure S10 *Inhibition ofCOX-1 by rofecoxib analogues* ***4a-c****,* ***5*** *and* ***6****. Error bars indicate standard error mean (SEM) obtained from two measurements at each concentration.*

3. Cytotoxicity assays

3.1. Reagents and cells.

Reagents were obtained from Sigma (St. Louis, MO) unless indicated otherwise. Annexin V-FITC (AnnV) was acquired from Biotium (Hayward, CA) and Apostat from R&D (R&D Systems, Minneapolis, MN USA). All cancer cell lines (A375, B16, B16F10, 518A2, HCT116, SW480, SW620 and CT26CL25) were cultured in HEPES (4-(2-hydroxyethyl)-1-piperazineethanesulfonic acid)-buffered RPMI-1640 medium supplemented with 10% FCS (fetal calf serum), 2 mM L-glutamine, 0.01% sodium pyruvate and antibiotics (culture medium) at 37 °C in a humidified atmosphere with 5% CO_2. After being detached by trypsinization, cells were seeded in defined number as follows: 2.5×10^3 cells/well in 96-well plates for viability assessment and 2×10^4 cells/well in 24-well plates for flow cytometry. Peritoneal resident macrophages were taken from C57BL/6 mice, obtained from the animal facility at the Institute for Biological Research "Siniša Stanković" by peritoneal lavage with an ice-cold phosphate buffer solution (PBS). The handling of animals and protocol for obtaining cells is in agreement with the rules of the European Union and approved by the Institutional Animal Care and Use Committee at IBISS (02-09/16). Cells were counted and seeded in 96-well plates and incubated overnight. Before treatment, non-adherent cells were removed. Compounds **4a-c**, **5** and **6** were dissolved in DMSO and stored at −20 °C for a month. Working solutions were freshly prepared in 10% FCS-RPMI-1640. Controls were treated with culture medium containing the equivalent amount of DMSO.

3.2. Cell viability tests.

The number of viable cells in culture was estimated by the MTT (3-(4,5-dimethylthiazol-2-yl)-2,5-diphenyltetrazolium bromide) and CV (crystal violet) tests. Cells were incubated in the presence of compounds **4a-c**, **5** and **6** for 48 h, then the viability was measured as described.[4]

The results were expressed as a percentage of a value obtained for the untreated control cells that was arbitrarily set to be 100%. A non-linear regression analysis of obtained results was done by using GraphPad Prism software to calculate IC_{50} values.

3.3. AnnexinV-FITC/PI and caspase detection.

A375 cells were treated with **5** for 48 h, trypsinized, and stained with AnnexinV-FITC (Ann) or propidium iodide (PI), or apostat as proposed by the manufacturer. Cells were analyzed by CyFlow® Space Partec using the PartecFloMax® software.

3.4. Cell staining with CFSE.

To quantify cell division rate, cells were incubated for 10 min at 37 °C in the presence of CFSE (1 μM) and upon removal of dye, cells were exposed to an IC_{50} dose of **5** for 48 h and subsequently quantified using flow cytometry.

3.5. Wound healing test.

Cell motility and proliferation was analyzed using wound healing assay. A375 and 518A2 cells were cultivated in 6-well plates until 80% of confluence in monolayers was reached. The scratch was made using a sterile pipette tip which was drawn across the center of the wells. The scratched cell layers were washed twice with fresh PBS to remove loose or damaged cells. Cells were than cultivated in the presence of an IC_{50} dose of **5** for 48 h, washed and finally fixed with 4% paraformaldehyde (PFA) for 10 min at room temperature. The wounds were digitally photographed using a Nikon inverted microscope TS2 with 5 MPIX camera.

3.6. Measurement of intracellular nitric oxide

The intracellular dye 4-amino-5-methylamino-2′,7′-difluorofluorescein diacetate (DAF, 5 μM) was used for quantification of NO. After treatment for 48 h, the cells were incubated in RPMI, without phenol red, for 1 h at 37 °C. Subsequently the dye (DAF) was removed and the cells where additionally incubated for 15 min in serum/phenol red free conditions, with an aim to complete de-esterification of intracellular diacetates. Finally, cells were resuspended in PBS and analyzed as described.[4]

3.7. Measurement of intracellular reactive oxygen (ROS) and nitrogen (RNS) species

A375 cells were stained with 1 μM of dihydrorhodamine-123 (DHR) for 20 min prior to exposure to an IC_{50} dose of **5**. After 48 h, the cells were trypsinized, washed and re-suspended in PBS, then analyzed with CyFlow® Space Partec using the PartecFloMax®.

3.8. Nitrite detection

Nitrite accumulation, an indicator of NO production, was estimated in the cellular supernatants after 48 h of cultivation in the presence of 5 by the Griess reaction.[5] In order to detect spontaneous release of nitric oxide, **5** was diluted in the culture medium or conditioned medium; conditioned medium is culture medium collected from cell cultures after 72 h of incubation. An equal volume of culture supernatants were mixed with an equal volume of the Griess reagent prepared from 0.1% N-(1-naphthyl)ethylenediamine dihydrochloride and 1% sulfanilamide in 5% H_3PO_4, mixed at a 1:1 ratio. Plates were incubated at room temperature for 10 min and the absorbance of the developed dye was measured at 570 nm in a DV 990 BV6 microplate reader (GCD multiplate reader, Roma, Italy). The nitrite concentration was calculated from a $NaNO_2$ standard curve.

3.9. Western blot.

Cells were lysed in protein lysis buffer (62.5 mM Tris-HCl pH 6.8, 2% w/v sodium dodecyl sulfate (SDS), 10% glycerol, 50 mM dithiothreitol) and lysates were electrophoretically separated on 12% SDS-polyacrylamide gels. PageRuler prestained ladder (Thermo Fisher Scientific; Waltham, MA, USA) was used as a protein molecular weight marker. Transfer of protein on polyvinylidene difluoride membranes was done at 5 mA/cm^2 with a semidry blotting system (Fastblot B43, BioRad; Göttingen, Germany). Blocking of membranes was done with 5 % Blotto (non-fat dry milk). Membranes were incubated overnight at 4°C with specific primary antibodies to COX-1, COX-2 (both from Santa Cruz Biotechnology; Dallas, TX, USA), β actin (Sigma–Aldrich, St. Louis, MO, USA) while the goat anti-rabbit IgG- horseradish peroxidase (Cell Signaling Technology, Danvers, MA, USA), donkey anti-goat IgG horseradish peroxidase (Abcam, Cambridge, UK), goat anti-mouse IgG horseradish peroxidase (Invitrogen, Carlsbad, CA, USA) were used as secondary antibodies. Bands were visualized by a chemiluminescence detection system (ECL, GE Healthcare; Chalfont St. Giles, Buckinghamshire, UK). All proteins were detected from the same membrane. For re-staining membranes were stripped with O.2M NaOH 10 min.

3.10. Statistics

Cells were treated with a range of concentrations in triplicate; IC_{50} concentrations were calculated from at least three independent experiments. Significance of the differences between various treatments was calculated by the analysis of variance (ANOVA), followed by the Student-Newman-Keuls test. A p value less than 0.05 was considered significant.

3.11. Molecular docking studies

Ligand structures were constructed with Avogadro 1.1.1.[6] Ligand geometries were optimized with the xtb code[7] using the GFN2 parameter set. The atomic partial charges for each ligand

were derived at the HF/6-31+G* level according to the RESP[8-11] procedure using Gaussian 09[12] and antechamber. The COX-2 crystal structure PDB ID: 4Z0L[17] was downloaded from www.rcsb.org.[13] All ligands and non-standard residues except for the heme groups, and all water molecules were removed with the UCSF Chimera package.[14] Ligand molecules and one monomer of COX-2 was prepared for docking with AutoDock Tools 1.5.6[15] according to a protocol published earlier.[16] Docking was performed with AutoDock 4.2.5.1[15] following a protocol published earlier.[16]

Table S10 *IC_{50} values for the cytotoxicity of **4a-c**, **5** and **6** for isolated macrophages measured by the crystal violet (CV) test.*

Compound	IC_{50} (μM)
**4a**	48.8±1.20
**4b**	65.7±5.75
**4c**	66.15±1.91
**5**	37.1±1.73
**6**	/

*Peritoneal resident macrophages were taken from C57BL/6 mice, obtained from the animal facility at the Institute for Biological Research "Siniša Stanković" by peritoneal lavage with a ice-cold phosphate buffer solution (PBS).

Table S11 *Selectivity index values of celecoxib in melanoma and colon cancer cell lines.*

Cell line	Selectivity index				
	**4a**	**4b**	**4c**	**5**	**6**
A375	3.1	4.7	6.3	7.9	/
518A2	2.3	4.5	1.6	6.7	/
B16	1.6	3.0	2.1	3.2	/
B16F10	1.9	2.5	2.5	4.8	/
HCT116	3.8	1.8	3.4	3.7	/
CT26CL25	1.3	1.8	2.3	1.3	/
SW480	2.6	1.8	2.5	4.3	/
SW620	2.0	3.0	1.5	3.5	/

*The selectivity index was calculated as a ratio between the IC_{50} of the compounds cytotoxicity determined for macrophages and the IC_{50} of the compounds cytotoxicity determined for the given cell line (IC_{50} macrophages / IC_{50} cancer). No value was calculated for **6**, as it showed no effect on the viability of the tested cell lines up to 50 μM

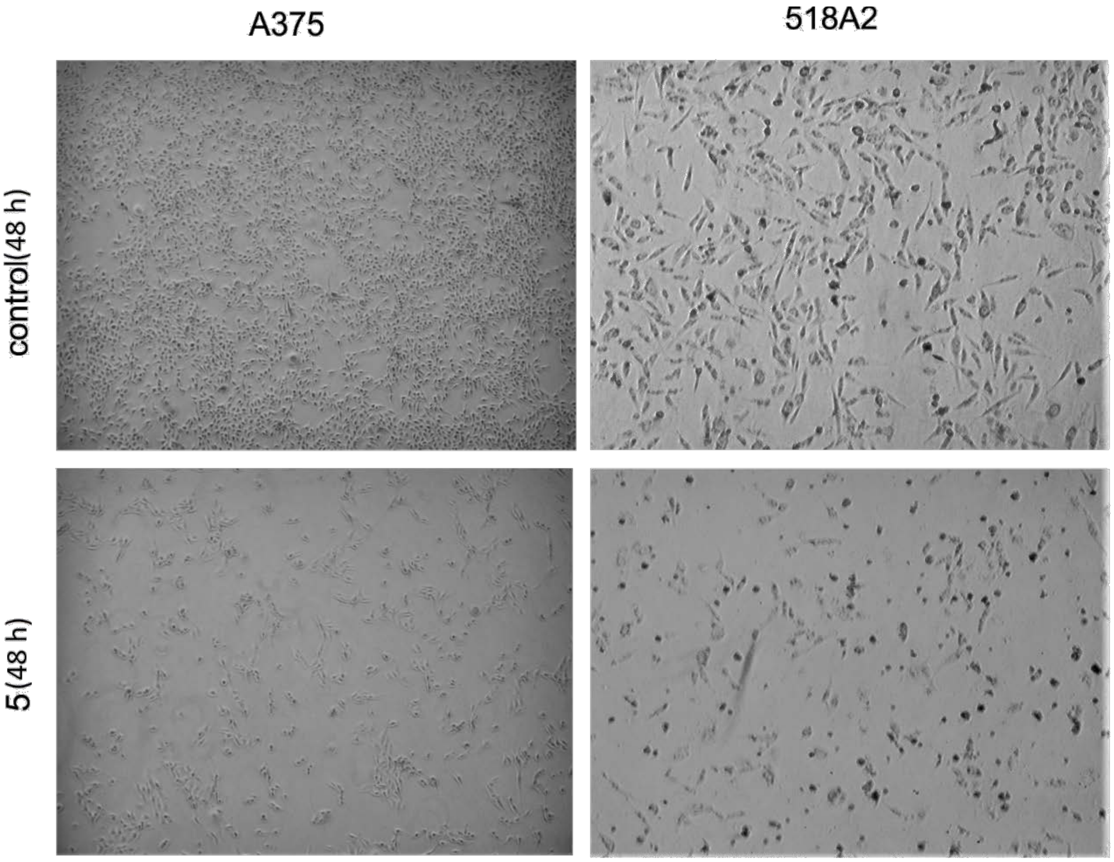

Figure S11 *Light microscopy of cells after incubation with* ***5*** *for 48 h; magnification x40. A representative out of three independent experiments was presented*

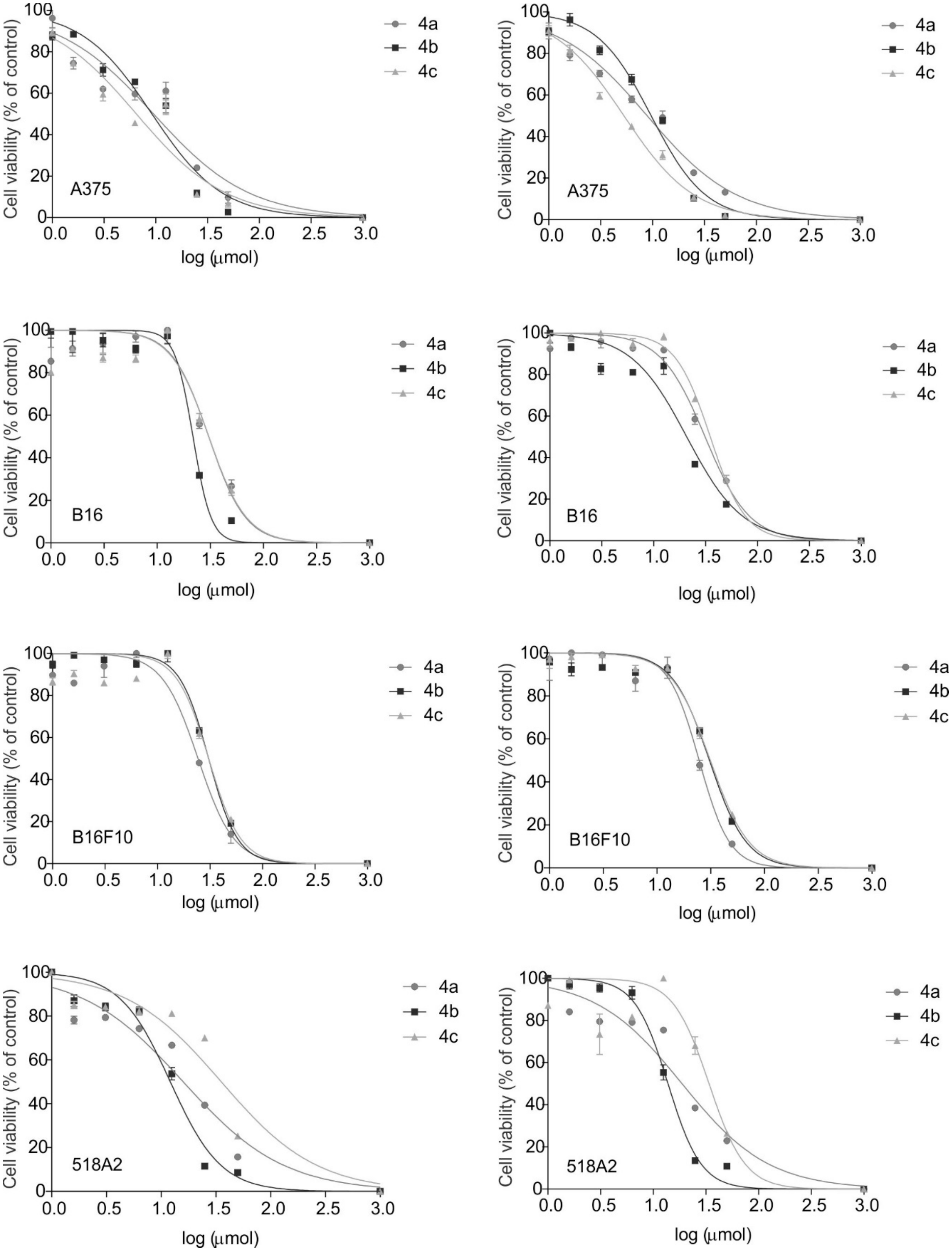

Figure S12 *Influence of rofecoxib analogues* ***4a-c*** *on melanoma viability***.** *A375, 518A2, B16, B16F10 cells were exposed to* ***4a-c*** *for 48 h and the number of viable cells was analyzed using MTT (left panel) and CV (right panel). The data are presented as percentage of control ± SD from one representative out of three independent experiments. * p<0.05 refers to untreated cultures.*

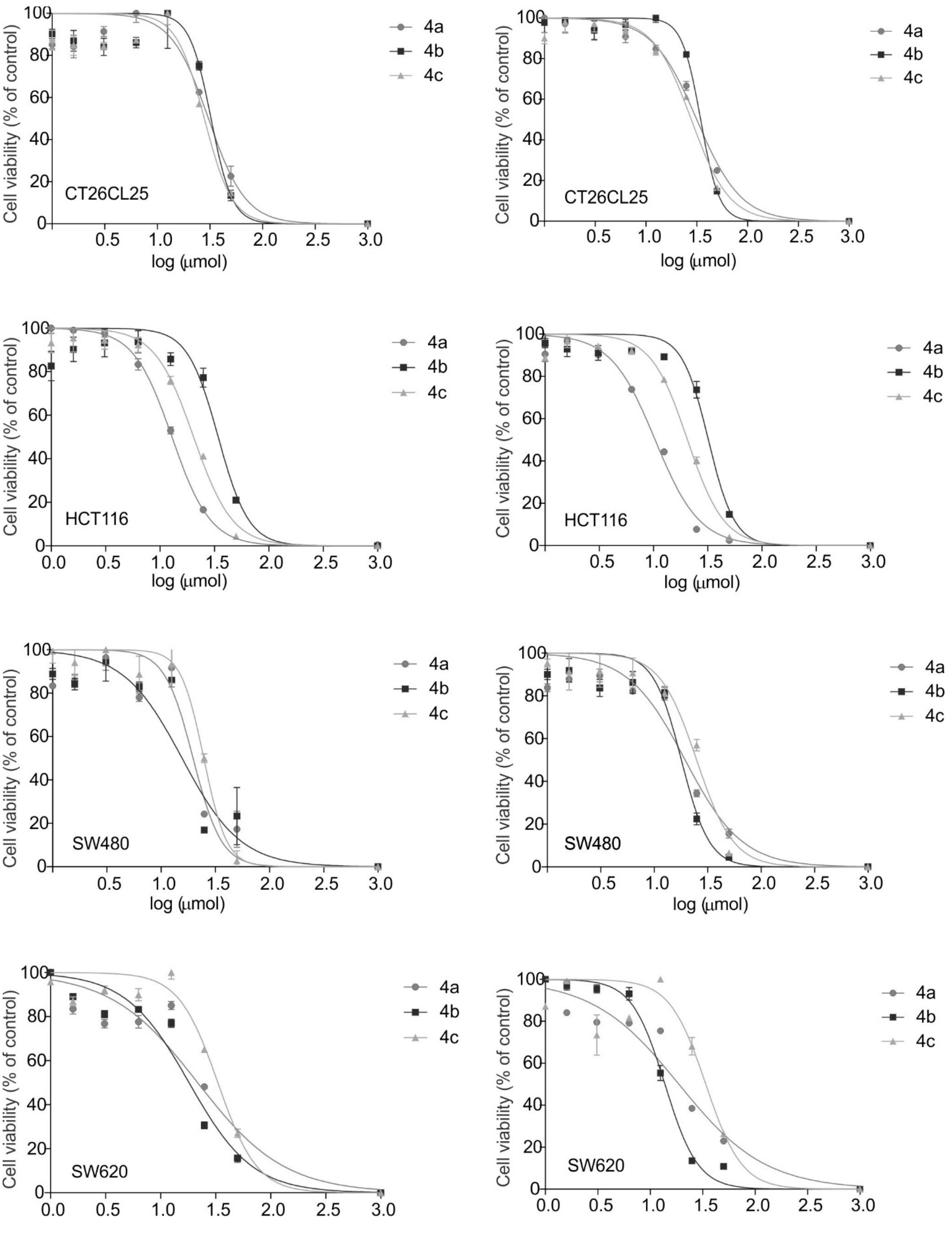

Figure S13 *Influence of rofecoxib analogues **4a-c** on viability of colon cancer cell lines. CT26CL25, HCT116, SW620 and SW480 cells were exposed to **4a-c** for 48 h and the number of viable cells was analyzed using MTT (left panel) and CV (right panel). Results were presented as Mean* ± *SV from three independent experiments. * p<0.05 refers to untreated cultures.*

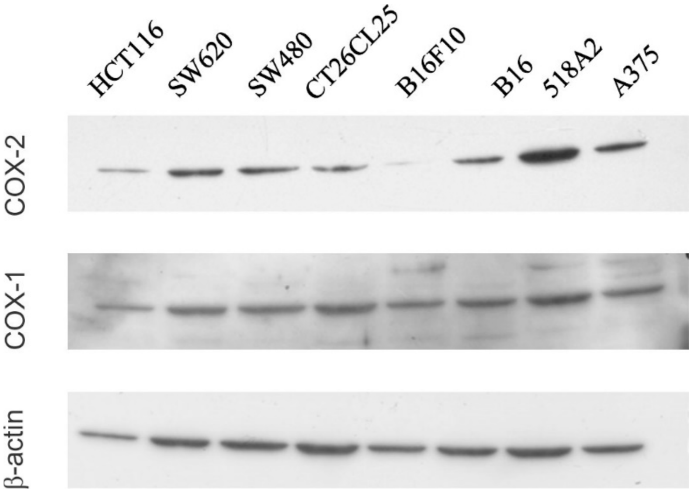

Figure S14 *COX-1 and COX-2 expression. The expression of COX enzymes was evaluated by WB. Full-length blots are presented (COX-1-72 kDa, COX-2-70-72 kDa, b-actin 42kDa).*

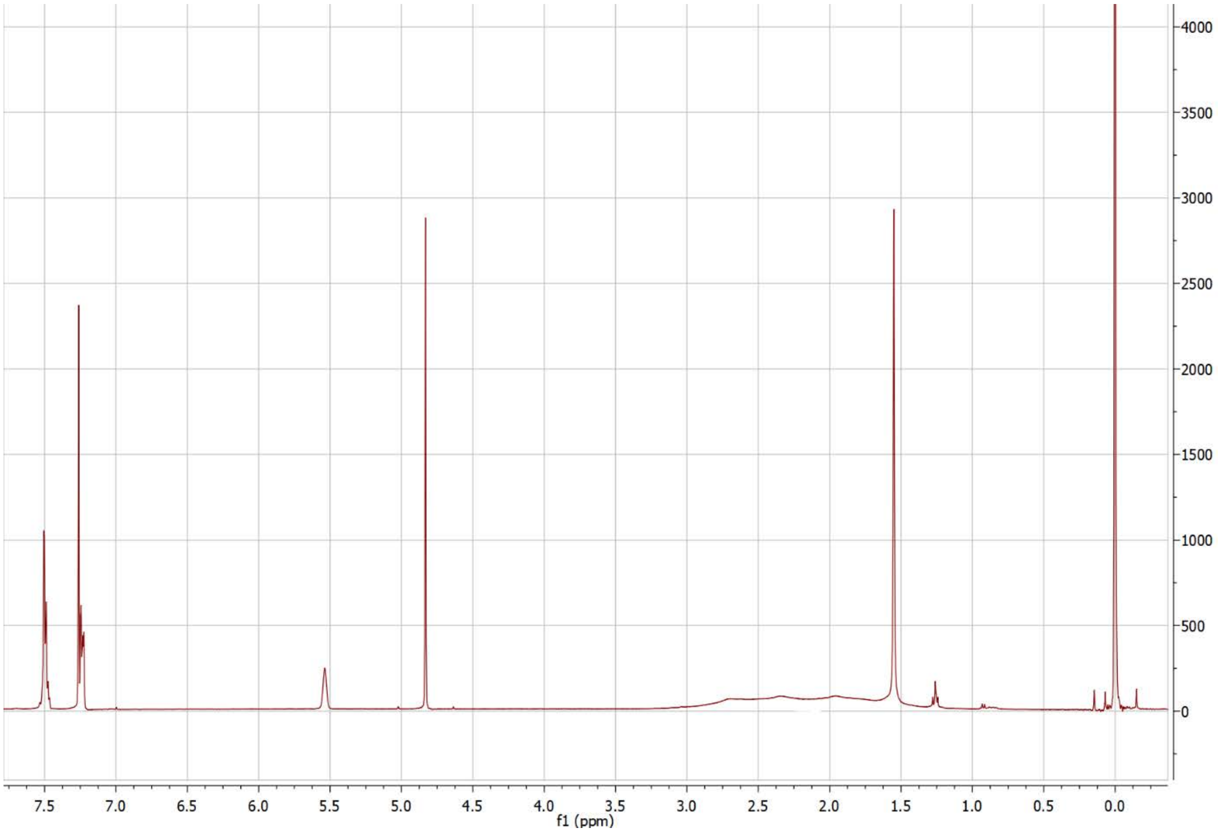

*^{1}H NMR spectrum of compound **4a***

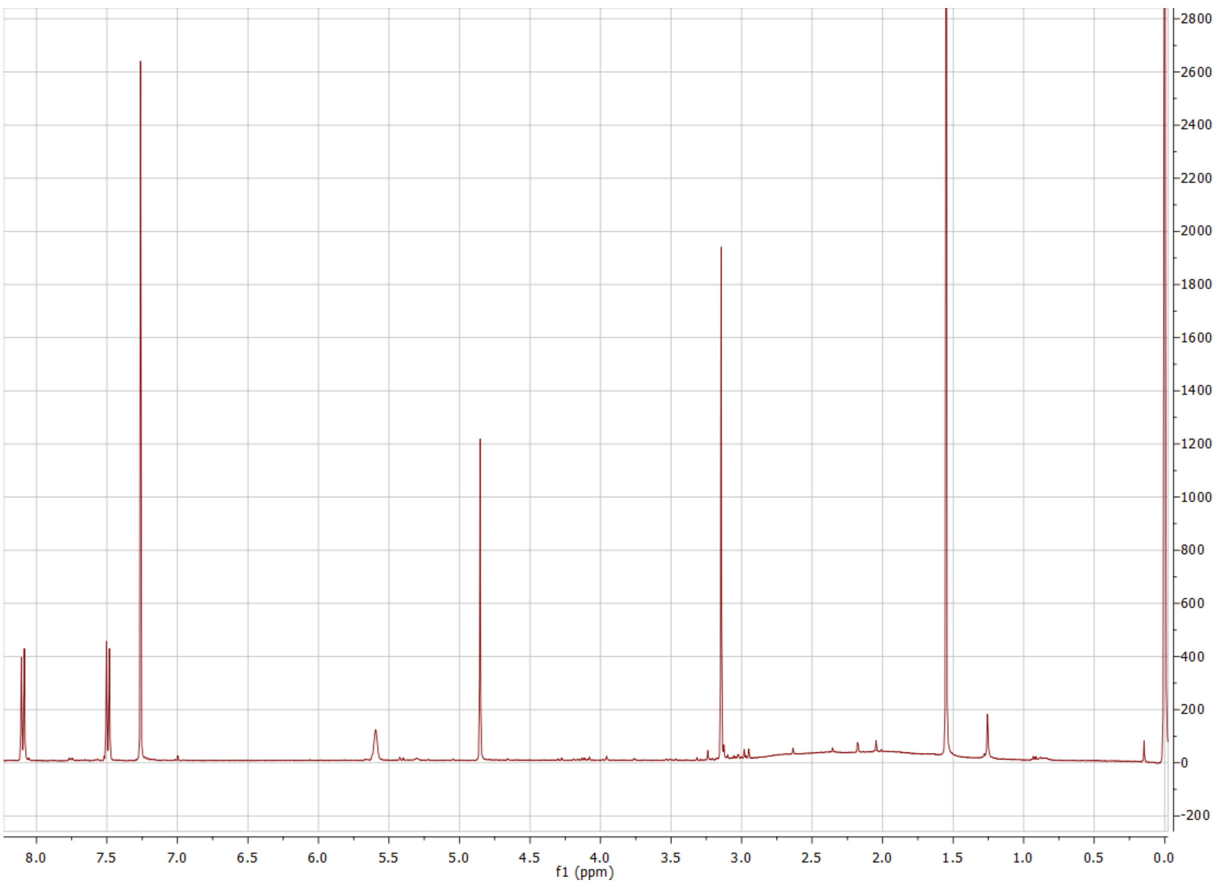

*^{1}H MNR spectrum of compound **4b***

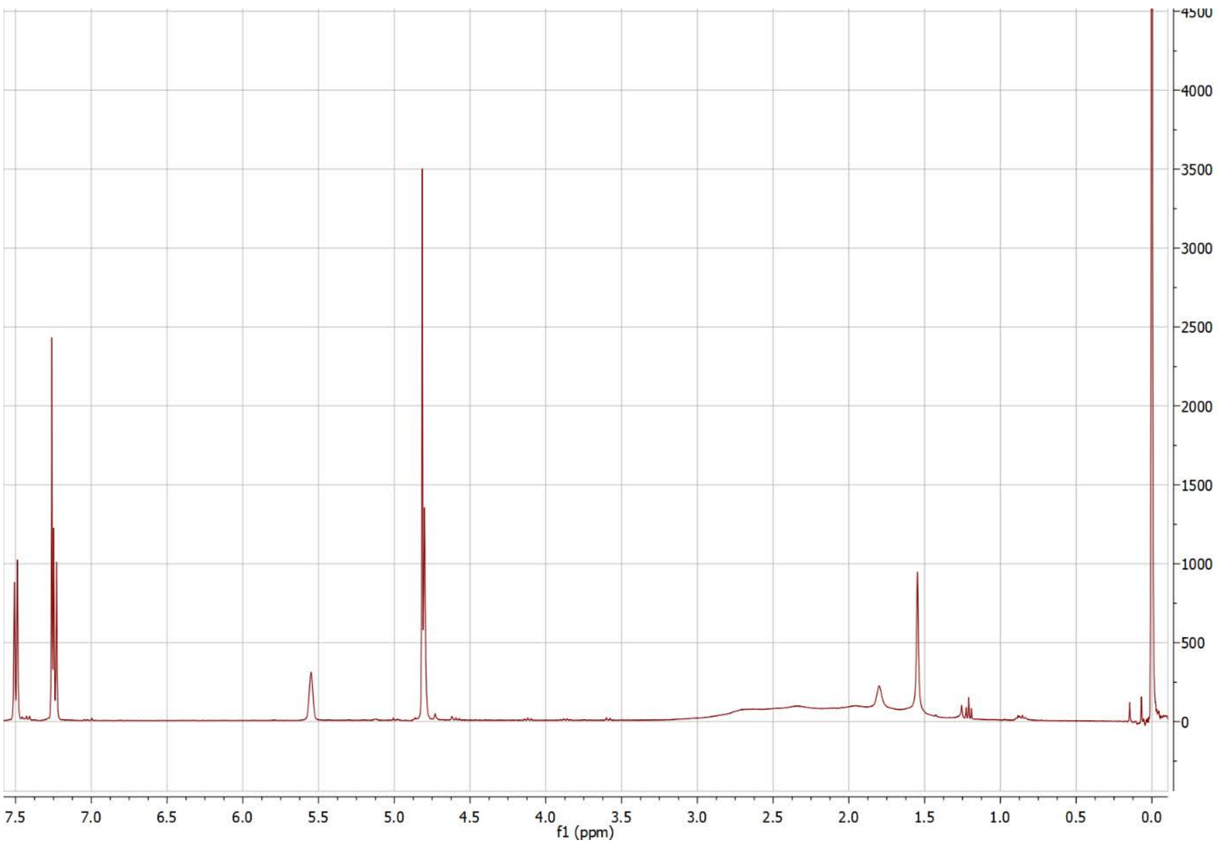

*^{1}H NMR spectrum of compound **4c***

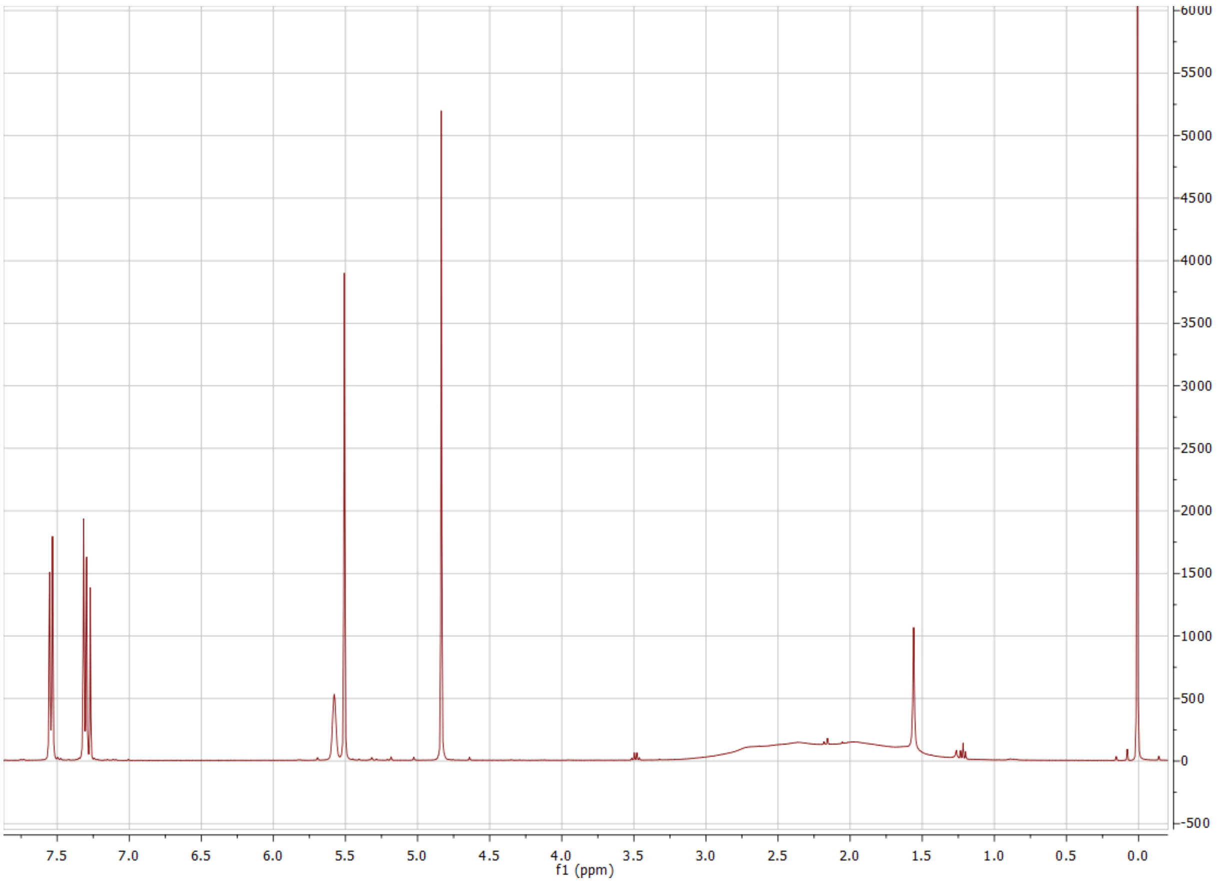

^{1}H NMR spectrum of compound ***5***

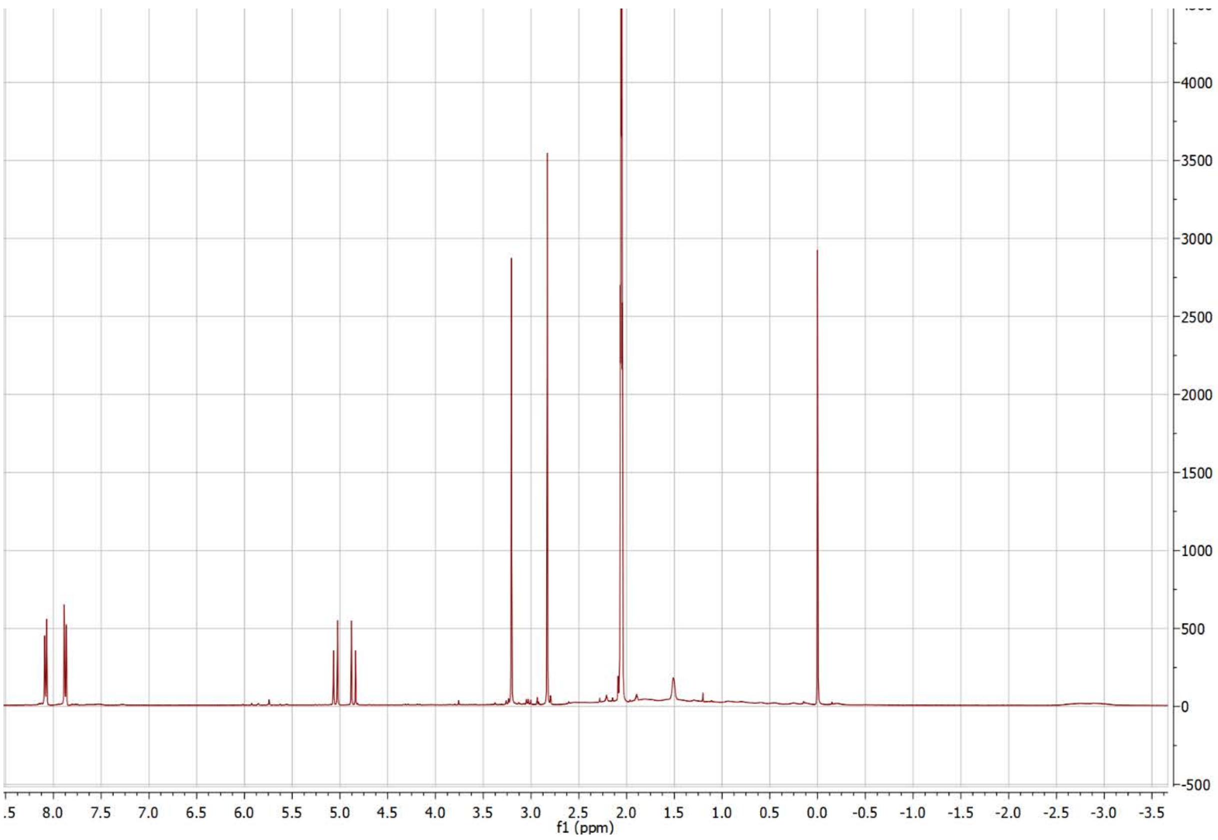

^{1}H NMR spectrum of compound ***6***

References:

1. CrysAlis Pro: Data collection and data reduction software package, Rigaku Inc.
2. Sheldrick, G.M., SHELXT - Integrated space-group and crystal-structure determination *Acta Cryst.* **A71**, 3-8(2015)
3. Sheldrick, G.M., Crystal structure refinement with SHELXL *Acta Cryst.* **C71**, 3-8 (2015)
4. Mijatovic, S.; Maksimovic-Ivanic, D.; Radovic, J.; Miljkovic, D.; Harhaji, L.; Vuckovic, O.; Stosic-Grujicic, S.; Mostarica Stojkovic, M.; Trajkovic, V., *Cell. Mol. Life Sci*. **2005**, 62 (5), 589-98.
5. Mijatovic, S. *et al.* Anti-glioma action of aloe emodin: the role of ERK inhibition. *Cell. Mol. Life Sci.* **62**, 589-598 (2005).

6. Green, L. C. *et al.* Analysis of nitrate, nitrite, and [15N]nitrate in biological fluids. *Anal. Biochem.* **126**, 131-138 (1982).

7. Hanwell, M. D. *et al.* Avogadro: an advanced semantic chemical editor, visualization, and analysis platform. *J Cheminform* **4**, 1758-2946 (2012).

8. Grimme, S., Bannwarth, C. & Shushkov, P. A Robust and Accurate Tight-Binding Quantum Chemical Method for Structures, Vibrational Frequencies, and Noncovalent Interactions of Large Molecular Systems Parametrized for All spd-Block Elements (Z = 1–86). *J. Chem. Theory Comput* **13**, 1989-2009, (2017).

9. Singh, U. C. & Kollman, P. A. An approach to computing electrostatic charges for molecules. *J. Comput. Chem.* **5**, 129-145, (1984).

10. Besler, B. H., Merz, K. M. & Kollman, P. A. Atomic charges derived from semiempirical methods. *J. Comput. Chem.* **11**, 431-439, (1990).

11. Bayly, C. I., Cieplak, P., Cornell, W. & Kollman, P. A. A well-behaved electrostatic potential based method using charge restraints for deriving atomic charges: the RESP model. *J. Phys. Chem.* **97**, 10269-10280, (1993).

12. Frisch, M. J. *et al. Gaussian 09 Revision A.02*. (Gaussian Inc. Wallingford CT 2009, 2009).

13. Berman, H. M. *et al.* The Protein Data Bank. *Nuc. Ac. Res.* **28**, 235-242 (2000).

14. Pettersen, E. F. *et al.* UCSF Chimera--a visualization system for exploratory research and analysis. *J. Comput. Chem.* **25**, 1605-1612 (2004).

15. Morris, G. M. *et al.* AutoDock4 and AutoDockTools4: Automated docking with selective receptor flexibility. *J. Comput. Chem.* **30**, 2785-2791, (2009).

16. Sárosi, M.-B., Neumann, W., Lybrand, T. P. & Hey-Hawkins, E. Molecular Modeling of the Interactions between Carborane-Containing Analogs of Indomethacin and Cyclooxygenase-2. *J. Chem. Inf. Model.* **57**, 2056-2067, (2017).

17. Neumann, W. *et al. nido*-Dicarbaborate Induces Potent and Selective Inhibition of Cyclooxygenase-2. *ChemMedChem* **11**, 175–178, (2016)
